# Body Mass Index, Clinical Outcomes, and Mortality in Heart Failure: A Mendelian Randomization Study

**DOI:** 10.1016/j.jacc.2026.02.5093

**Published:** 2026-06-02

**Authors:** Nicholas Sunderland, Geraldine Asselin, Albert Henry, Christopher P. Nelson, Louis-Philippe Lemieux Perreault, Folkert W. Asselbergs, Eric Boersma, Thomas P. Cappola, Olympe Chazara, William Chutkow, Marie-Christyne Cyr, Apostolos Gkatzionis, Hongsheng Gui, Carolina Haefliger, Åsa K. Hedman, Hans Hillege, Craig L. Hyde, Frederick K. Kamanu, Isabella Kardys, Andrea L. Koekemoer, William E. Kraus, Chim C. Lang, Anders Malarstig, Kenneth B. Margulies, Nicholas A. Marston, Giorgio E.M. Melloni, Michael P. Morley, Michelle L. O'Donoghue, Anjali T. Owens, Dirk S. Paul, Kate Tilling, Pim van der Harst, Jessica van Setten, Marion van Vugt, Niek Verweij, Abirami Veluchamy, Lars Wallentin, Xiaosong Wang, Heming Xing, Yifan Yang, Harvey D. White, Faiez Zannad, J. Gustav Smith, Hans-Peter Brunner-La Rocca, David E. Lanfear, Douglas L. Mann, Simon de Denus, Jean-Claude Tardif, Adriaan A. Voors, Nilesh J. Samani, Patrick T. Ellinor, Christian T. Ruff, Marc S. Sabatine, Naveed Sattar, John J.V. McMurray, Lavinia Paternoster, Marie-Pierre Dubé, R. Thomas Lumbers

**Affiliations:** a MRC Integrative Epidemiology Unit, Bristol Medical School, University of Bristol, Bristol, United Kingdom; b NIHR Bristol Biomedical Research Centre, University Hospitals Bristol and Weston NHS Foundation Trust and University of Bristol, Bristol, United Kingdom; c Montreal Heart Institute, Pharmacogenomics Centre, Montréal, Québec, Canada; d Institute of Cardiovascular Science, University College London, London, United Kingdom; e Department of Cardiovascular Sciences, University of Leicester, Leicester, United Kingdom; f NIHR Leicester Biomedical Research Centre, Glenfield Hospital, Leicester, United Kingdom; g Department of Cardiology, Amsterdam Cardiovascular Sciences, Amsterdam University Medical Center, University of Amsterdam, Amsterdam, the Netherlands; h The National Institute for Health and Care Research University College London Biomedical Research Centre, University College London, London, United Kingdom; i Institute of Health Informatics, University College London, London, United Kingdom; j Department of Cardiology, Cardiovascular Institute, Erasmus MC, University Medical Center, Rotterdam, the Netherlands; k Penn Cardiovascular Institute, Perelman School of Medicine, University of Pennsylvania, Philadelphia, Pennsylvania, USA; l Centre for Genomics Research, Discovery Sciences, BioPharmaceuticals R&D, AstraZeneca, Cambridge, United Kingdom; m Novartis Institutes for Biomedical Research, Cambridge, Massachusetts, USA; n Center for Individualized and Genomic Medicine Research, Department of Internal Medicine, Henry Ford Hospital, Detroit, Michigan, USA; o Cardiovascular Medicine Unit, Department of Medicine Solna, Karolinska Institute, Stockholm, Sweden; p Department of Cardiology, University Medical Center Groningen, University of Groningen, Groningen, the Netherlands; q Pfizer Worldwide Research & Development, Cambridge, Massachusetts, USA; r TIMI Study Group, Division of Cardiovascular Medicine, Brigham and Women's Hospital, Harvard Medical School, Boston, Massachusetts, USA; s Duke University School of Medicine, Durham, North Carolina, USA; t Division of Cardiovascular Research, University of Dundee, Dundee, United Kingdom; u Tuanku Muhriz Royal Chair, National University of Malaysia, Bangi, Malaysia; v Department of Cardiology, University Medical Center Utrecht, Utrecht, the Netherlands; w Division of Molecular and Clinical Medicine, University of Dundee, Ninewells Hospital and Medical School, Dundee, United Kingdom; x Department Medical Sciences and Uppsala Clinical Research Center, Uppsala University, Uppsala, Sweden; y Sanofi Research, Cambridge, Massachusetts, USA; z Health New Zealand Te Whatu Ora, Te Toka Tumai Auckland, Green Lane Cardiovascular Service, Auckland City Hospital, Auckland, New Zealand; aa Universite de Lorraine, CHRU Nancy, Inserm, CIC, Nancy, France; bb Department of Molecular and Clinical Medicine, Institute of Medicine, Gothenburg University and Sahlgrenska University Hospital, Gothenburg, Sweden; cc Science for Life Laboratory, Gothenburg University, Gothenburg, Sweden; dd Department of Cardiology, Clinical Sciences, Lund University and Skåne University Hospital, Lund, Sweden; ee Wallenberg Center for Molecular Medicine and Lund University Diabetes Center, Lund University, Lund, Sweden; ff Cardiovascular Research Institute (CARIM), Maastricht University, Maastricht, the Netherlands; gg Center for Individualized and Genomic Medicine Research, Henry Ford Health, Detroit, Michigan, USA; hh Center for Cardiovascular Research, Washington University School of Medicine, St Louis, Missouri, USA; ii Montreal Heart Institute, and Faculty of Pharmacy, Université de Montréal, Montréal, Québec, Canada; jj Montreal Heart Institute, and Faculty of Medicine, Université de Montréal, Montrėal, Québec, Canada; kk Cardiac Arrhythmia Service and Cardiovascular Research Center, Massachusetts General Hospital, Cambridge, Massachusetts, USA; ll School of Cardiovascular and Metabolic Health, University of Glasgow, Glasgow, United Kingdom; mm Montreal Heart Institute, Pharmacogenomics Centre, and Faculty of Medicine, Université de Montréal, Montréal, Québec, Canada

**Keywords:** heart failure, obesity, genome-wide association study, Mendelian randomization, index-event bias

## Abstract

**Background:**

Excess adiposity, most commonly indexed through body mass index (BMI), is strongly associated with the development of heart failure (HF). Weight loss therapies improve outcomes in patients with obesity and HF with preserved left ventricular ejection fraction (LVEF), but their effects in HF with reduced LVEF remain unclear.

**Objectives:**

The aim of this work is to determine whether higher BMI is associated with adverse clinical outcomes in patients with HF and whether there is effect modification by LVEF subgroup.

**Methods:**

Two-sample Mendelian randomization (MR) was used, with genome-wide significant loci associated with BMI as instrumental variables and outcome data from a genome-wide association study (GWAS) of time-to-event clinical outcomes in patients with HF. A total of 50,636 individuals of European ancestry with established HF from 22 cohorts were included in the genetic analysis: 12 HF trials, 1 prospective case-cohort study, 9 cohorts nested within non-HF cardiovascular trials, and 1 population-based cohort derived from the UK Biobank.

The exposure was genetically predicted BMI and the outcome measures were all-cause mortality and a composite of cardiovascular mortality or HF hospitalization. Genetic associations for the outcomes were derived from our GWAS and MR was used to estimate the unbiased association of genetically predicted BMI with these clinical outcomes.

**Results:**

The mean BMI was 29.2 ± 5.8 kg/m^2^. Over a median follow-up of 27.0 months, all-cause mortality occurred in 11,454 patients (23%), and 11,360 participants (22%) experienced the composite endpoint. Genetically predicted BMI was associated with an increased rate of both all-cause mortality (HR per SD [4.8 BMI units] 1.21; 95% CI: 1.13-1.29; *P* = 9 × 10^-8^) and the composite outcome (HR 1.29; 95% CI: 1.20-1.38; *P* = 8 × 10^-13^). Associations were consistent across LVEF ≤40% and >40%: for all-cause mortality, HR: 1.16 (95% CI: 0.99-1.37) and 1.20 (95% CI: 0.94-1.53); and for the composite outcome, HR: 1.30 (95% CI: 1.15-1.48) and 1.57 (95% CI: 1.29-1.91), respectively.

**Conclusions:**

Among patients with HF, higher BMI was associated with increased all-cause mortality and cardiovascular death or HF hospitalization, supporting the potential role of weight-management strategies across the ejection fraction spectrum.

## Graphical abstract

**Figure:**
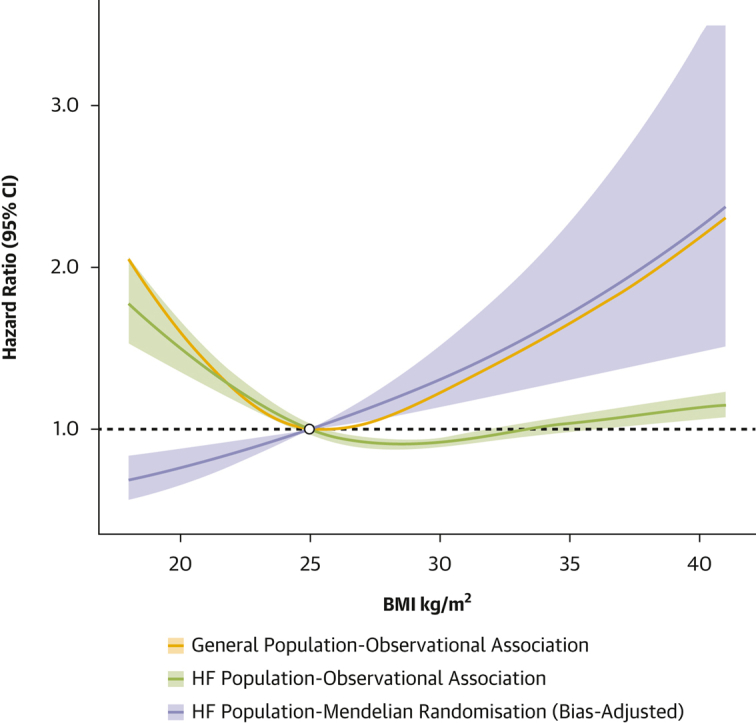

Heart failure (HF) is a growing public health concern, and obesity—defined as having a body mass index (BMI) (the weight in kilograms divided by the square of the height in meters) of 30.0 kg/m^2^ or more—is increasingly recognized as a major causal risk factor.^1^, ^2^, ^3^, ^4^ Incretin-based therapies, including GLP-1 and dual glucose-dependent insulinotropic polypeptide/glucagon-like peptide (GIP/GLP)-1 receptor agonists, and—to a lesser extent—SGLT2 inhibitors, promote weight loss and reduce HF hospitalization in at-risk populations.^5^, ^6^, ^7^ Mendelian randomization (MR) studies further support a positive association between higher BMI and new-onset HF, regardless of whether left ventricular ejection fraction (LVEF) is reduced or preserved.^8^^,^^9^

**Flipping the Paradox: Unmasking the True Influence of Obesity on Heart Failure (HF) Prognosis**

**The Obesity Survival Paradox in HF**

Obesity is an established causal risk factor for the development of HF. Paradoxically, among patients with established HF, higher body mass index (BMI) appears to protect against mortality in clinical studies, a phenomenon termed the "obesity survival paradox." This observation has led to uncertainty about the role of weight reduction in patients with HF. Such paradoxical associations are common in prognosis research.

**Why Risk Factors Can Appear Protective After Disease Onset**

HF can arise from multiple risk factors such as obesity, coronary artery disease, and hypertension. In populations with established HF, associations between these risk factors can be distorted by index event bias (a form of collider bias); this bias can attenuate or even reverse risk-factor associations with prognosis. For example, obesity may appear protective in HF not because it improves outcomes but because affected individuals developed HF with fewer or less severe alternative risk factors that carry worse prognoses. Conventional covariate adjustment cannot correct for index-event bias and indeed may amplify it, and confounding and reverse causation can further bias prognostic associations.

**How Genetic Approaches Can Enable Unbiased Estimates of Risk Factors on Prognosis**

Genetic variants can be used as proxies for risk factors to estimate their effects on disease outcomes under the Mendelian paradigm, mitigating bias because of confounding and reverse causation. Genome-wide association studies (GWAS) of disease prognosis (the outcome) are susceptible to index event bias, but unlike conventional observational analyses, this bias can be modeled and corrected. Mendelian randomization can then be applied to estimate the effects of risk factors on prognosis, minimizing bias from confounding, reverse causation, and index-event bias.

**Broader Implications for Prognostic Research Across Clinical Medicine**

Paradoxical associations between risk factors and disease outcomes are common in prognosis research across clinical medicine, and often underappreciated. Genetic studies of disease progression can provide unbiased evidence on the influence of risk factors to inform clinically important questions about disease management.

However, the effect of intentional weight reduction on mortality among patients with established HF remains debated, partly because of the obesity survival paradox, in which observational studies report a protective association between higher BMI and mortality.^10^^,^^11^ This paradox may be explained by index event bias, in which higher BMI appears protective in observational studies because affected individuals have fewer or less severe alternative HF risk factors.^10^^,^^12^^,^^13^ Recent trials in patients with heart failure with preserved ejection fraction (HFpEF) have shown that GLP-1 receptor agonists reduce BMI, improve quality of life, and increase 6-minute walk distance,^14^ whereas GIP/GLP-1 receptor agonists have demonstrated improvements in clinical outcomes.^15^ Although subgroup analyses from the SELECT (Semaglutide Effects on Cardiovascular Outcomes in People With Overweight or Obesity) trial suggested that GLP-1 receptor agonists improve clinical outcomes in patients with both HFpEF and heart failure with reduced ejection fraction (HFrEF), the findings are debated, and uncertainty remains regarding the role of incretin-based therapies in patients with HFrEF with other studies showing signals for harm.^16^, ^17^, ^18^, ^19^, ^20^

Understanding the relationship between BMI and clinical outcomes in HFpEF and HFrEF is essential for determining the potential role of incretin-based therapies and other weight-loss interventions. MR uses genetic variation to estimate causal relationships, minimizing biases such as reverse causation, confounding, and misclassification.^21^ Applying this approach to estimate the association between BMI and clinical outcomes in HF offers an opportunity to provide evidence that may support the conduct of outcomes-driven randomized controlled trials of incretin-based therapies and other interventions aimed at reducing BMI.

In this study, we investigate the impact of higher BMI on clinical outcomes in HF, using genetic approaches to minimize bias to test the hypothesis that its true effects are adverse. First, we recapitulate the obesity survival paradox in HF using observational data from the UK Biobank. Next, we perform a time-to-event genome-wide association study (GWAS) of clinical outcomes stratified by LVEF and apply methods to account for bias inherent to prognostic study design. Finally, we use MR to estimate the association of genetically predicted higher BMI with all-cause mortality and a composite of cardiovascular (CV) mortality or HF hospitalization in patients with HF.

## Methods

### Observational association between BMI and HF: the paradox

#### Data source

The UK Biobank is a prospective cohort study of >500,000 UK residents aged between 40 and 69 years old and recruited between 2006 and 2010. The project has curated detailed health and genetic information from the participants alongside linked discharge data from secondary care (Hospital Event Statistics [HES]), and mortality data.^22^

#### Procedures and outcomes

HF was defined by the occurrence of any HF diagnosis code in the electronic health records: International Classification of Disease (ICD)-10 codes I110, I130, I132, I50∗; and ICD9 codes 428∗, 40201, 40211, 40291, 40401, 40403, 40411, 40413, 40491, 40493, as well as UK Biobank self-reported illness code 1076.^8^ HF admission followed the National Institute for Cardiovascular Outcomes Research (NICOR) definition of an unscheduled admission, of >1 day in duration, with primary diagnosis code for HF (defined above) and/or 4254/I255 (ischemic cardiomyopathy), 4251/I420 (dilated cardiomyopathy), or 4254/I429 (cardiomyopathy, unspecified).^23^ CV death was defined as any death resulting from HF, acute myocardial infarction (MI), stroke, or death due to other CV causes. Clinical outcome endpoints were defined according to the 2013 European Society of Cardiology Heart Failure Association guidelines^24^ and the 2014 American College of Cardiology/American Heart Association (ACC/AHA) Key Data Elements and Definitions for Cardiovascular Endpoint Events in Clinical Trials.^25^

#### Study population

Individuals enrolled in the UK Biobank (N = 502,411) were considered for study inclusion. The following exclusions were made: 54 participants who withdrew consent, 3,107 with missing baseline BMI data, and 2,618 with prevalent HF at recruitment (including a 6-month lag period postrecruitment). After exclusions, 496,678 individuals were included in the analysis, 14,366 of whom were subsequently diagnosed with HF (Supplemental Table 1).

#### Statistical analysis

To illustrate the HF obesity survival paradox observed in observational data, we investigated the association between BMI and the clinical outcomes of interest in people with HF compared with the general UK Biobank population. BMI was modeled as a categorical variable with strata defined as underweight (<18.5 kg/m^2^), normal (18.5-24.9 kg/m^2^), overweight (25.0-29.9 kg/m^2^), class I obesity (30.0-34.9 kg/m^2^), class II obesity (35.0-39.9 kg/m^2^), and class III obesity (>40 kg/m^2^). In addition, we modeled BMI as a continuous variable by fitting a 4-knot restricted cubic spline with knots at the 5th, 25th, 75th, and 95th BMI percentiles.^26^

Survival analysis was conducted using Cox proportional hazards regression to estimate the hazard of all-cause death and the Fine-Gray method to estimate the subdistribution hazard of CV death or HF hospitalization, with non-CV death as a competing risk. Individuals with established HF at enrollment were excluded, and for cases of HF occurring during follow-up, the baseline (t = 0) was set as the date of HF diagnosis. Models were run with no adjustment; adjustment for sex and age at enrollment or HF diagnosis in HF cases; and adjusted for sex, age, smoking status (never, previous, current), and baseline atrial fibrillation (AF), diabetes mellitus (DM), chronic kidney disease (CKD), hypertension, hyperlipidemia, MI, stroke, and chronic obstructive pulmonary disease (COPD). The proportional hazards assumption was assessed and validated by the inspection of the scaled Schoenfeld residuals plotted against time.

### GWAS

#### Study population

Twenty-two cohorts participating in the Heart Failure Molecular Epidemiology for Therapeutic Targets (HERMES) Consortium, comprising 50,636 individuals with established HF of European ancestry, were included in the genetic study (Supplemental Table 2).^27^ Twelve were HF cohorts (trials or prospective research cohorts), 9 were nested HF cohorts within non-HF cardiovascular trials or cohorts, and 1 cohort was derived from individuals in the UK Biobank with genetic data. Study-level LVEF data were available for 16 studies. For subgroup meta-analyses, cohorts were dichotomized by LVEF: ≤40% (trial entry criterion LVEF ≤40% or study mean LVEF ≤35%; 11 studies; 12,608 patients; mean LVEF 30.6%) or LVEF >40% (trial entry criterion LVEF >40% or study mean LVEF >45%; 5 studies; 9,597 patients; mean LVEF 54.3%).

#### Procedures and outcomes

The majority of cohorts were HF clinical trials and used standard HF inclusion criteria to define cases (Supplemental Table 2). For the UK Biobank, HF was defined using the diagnostic codes detailed here. Two clinical endpoints were investigated in the genetic analyses: all-cause mortality and a composite endpoint of CV mortality or HF hospitalization, as defined here.

#### Statistical analysis

Procedures for genotyping, imputation, and quality control have been previously reported.^8^ For each HF cohort, GWAS of time-to-event outcomes were performed using Cox proportional hazards regression. An additive genetic model with adjustment for sex, age at study initiation, HF status at enrollment (prevalent or incident), and other study-specific covariates, including principal components for genetic ancestry, randomization arm, or recruitment center. For cases with prevalent HF at study enrollment, the date of study enrollment was set at the baseline (t = 0). For cases of HF occurring during follow-up, the baseline (t = 0) was set as the date of HF diagnosis, if available, or the date of the first recorded HF diagnosis. GWAS meta-analyses were performed using Genome-Wide Association Meta-Analysis (GWAMA [version 2.2.2]) with an inverse-variance weighted fixed-effects model.^28^ Genetic variants are reported for which results were derived from at least 2 studies, pruned for independence using PLINK 2.0 (*r*^*2*^ < 0.001, 10,000 kb) with a reference panel consisting of 10,000 unrelated European ancestry participants.^29^^,^^30^ We investigated whether genetic variants previously associated with new-onset HF were also associated with clinical outcomes in patients with established HF, using summary statistics from our previous work^8^ and those from a recent multiancestry meta-analysis of all-cause HF.^31^ This analysis compared the observed replication rate with the expected rate, adjusted for statistical power, as previously described.^32^

We used the recently developed bivariate MR with corrected weighted least squares (CWBLS) adjustment to assess and adjust for index-event bias in the association of genetic variants identified in the GWAS (Supplemental Methods).^33^^,^^34^ In further index-event bias sensitivity analyses we used the Dudbridge,^35^ standard CWLS,^34^ Slope-Hunter,^36^ and traditional 2-sample MR approaches,^37^ to estimate bias correction factors and analyzed the impact of these index-event bias adjustments on our primary MR analysis (Supplemental Methods).

### MR

#### Data sources

The genetic instrument for BMI was derived from genome-wide association summary statistics from a Genetic Investigation of Anthropometric Traits (GIANT) consortium meta-analysis of >700,000 individuals of predominantly European ancestry.^38^ The GWAS data sources are listed in Supplemental Table 3. All contributing studies received approval from local institutional review boards. Meta-analysis of summary-level GWAS data was conducted in accordance with guidelines for study procedures provided by the University College London (UCL) Research Ethics Committee.

#### Selection of instruments

Variants were selected based on genome-wide significance for BMI (*P* < 5 × 10^-8^) and pruned for independence using PLINK 2.0 (*r*^*2*^ < 0.001, 10,000 kb) with a reference panel consisting of 10,000 unrelated European ancestry participants from the UK Biobank (Supplemental Table 4).^29^^,^^30^ To confirm the validity of this instrument in patients with established HF, we estimated the associations of the included variants in a GWAS of BMI in individuals with HF compared with non-HF controls in UK Biobank, testing for heterogeneity using Cochrane’s Q statistic.

#### Statistical analysis

MR was performed in a 2-sample framework, using the MendelianRandomization R package.^39^ The genetic instrument used in MR should satisfy 3 core instrumental variable assumptions. 1) Relevance: each variant is robustly associated with BMI, confirmed through genome-wide significance. 2) Independence: there are no measured or unmeasured confounders of the instrument and the outcome. 3) Exclusion restriction: the variants affect outcomes only through BMI, not via alternative biological pathways. The primary analysis used an inverse variance weighted (IVW) method, with sensitivity analyses conducted using MR Egger, weighted median, and weighted-mode methods designed to address the core MR assumptions, accounting for pleiotropy and imperfect instruments. The MR estimates represent the change in log hazard for HF clinical outcomes per standard deviation increase in BMI. The Z-test was used to assess for interaction between the LVEF stratified MR estimates.

## Results

### Recapitulating the obesity survival paradox in HF

**Figure 1:**
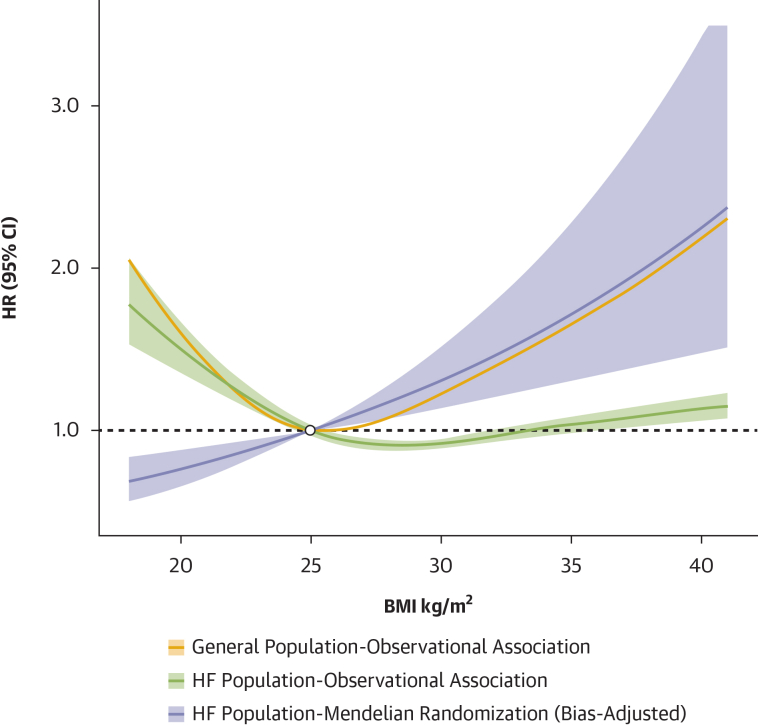
Association Between BMI and Mortality: Observational and MR Analyses Association between BMI and all-cause mortality. The plot presents data from 2 separate of analyses: observational BMI restricted cubic spline survival models using nongenetic data from either all individuals (yellow curve), or individuals with HF (green curve), within the UK Biobank and the MR estimate of the association between BMI and HF mortality, log-linearly extrapolated assuming a constant HR per standard deviation increase in genetically predicted BMI (purple curve), adjusted for index-event bias using the primary bias adjustment method (Bivariate MR CWBLS). Observational curves are derived from the models adjusted for age and sex, with estimates shown at the median age and for male sex, consistent with the covariate adjustment used in the GWAS of time-to-event outcomes. The observational analyses capture non-linear relationships between measured BMI and outcomes, whereas MR estimates assume a linear relationship on the log-hazard scale based on genetic instruments. All HRs are referenced to BMI = 25 kg/m^2^ (open circle). Shaded areas represent 95% CIs. BMI = body mass index; CWBLS = corrected weighted least squares; GWAS = genome-wide association studies; HF = heart failure; MR = Mendelian randomization.

To demonstrate the survival paradox in HF, we first estimated the association between measured BMI and clinical outcomes using conventional observational analysis of data from 14,366 individuals with HF in the UK Biobank. Baseline demographics are presented in Supplemental Table 1. Our findings replicated the HF obesity survival paradox: compared with normal weight (18.5-24.9 kg/m^2^), patients with HF in the overweight (25.0-29.9 kg/m^2^) and obesity class I (30.0-34.9 kg/m^2^) categories had an apparent lower rate of all-cause mortality, contrasting with that seen in the total UK Biobank population, in which overweight and obesity were associated with higher mortality rates relative to normal weight (Figure 1, Supplemental Table 5). Interestingly, conventional covariate adjustment for common CV risk factors appeared to strengthen the inverse association of higher BMI with clinical outcomes in patients with HF (Supplemental Table 5).

### GWAS of clinical outcomes in HF

Next, to enable the MR study, we conducted a time-to-event GWAS meta-analysis of all-cause mortality and the composite of CV mortality or HF hospitalization in patients with established HF. We analyzed a population comprising 50,636 individuals with HF of European ancestry from 22 studies—all of whom had available genetic data—and follow-up data on the clinical outcomes of interest (Table 1, Supplemental Table 2). Over a median follow-up of 27.0 months (IQR: 17.2-35.3 months), 11,454 participants (23%) reached the endpoint of all-cause mortality, and 11,360 participants (22%) experienced the composite endpoint of cardiovascular death or hospitalization for HF (Table 2). The mean age of participants was 66 ± 9.5 years, with 68% male. The mean BMI was 29 ± 5.8 kg/m^2^. A high proportion had comorbidities at enrollment: 46% had a history of MI, 51% had AF, 79% had hypertension, and 34% had DM. Eleven studies were classified as LVEF ≤40% (12,608 patients) and 5 as LVEF >40% (9,597 patients) (Table 1).

**Table 1:** Demographics and Clinical Characteristics of the Participants at Baseline

	All (N = 50,636)	LVEF ≤40% (n = 12,608)	LVEF >40% (n = 9,597)
Number of studies	22	11	5
Study type			
RCT	26,514 (52)	7,495 (59)	9,207 (96)
Research cohort	6,981 (14)	5,113 (41)	390 (4)
Genomic biobank	17,141 (34)	0 (0)	0 (0)
Race or ethnic group			
White	50,636 (100)	12,608 (100)	9,597 (100)
BMI, kg/m^2^	29.2 ± 5.8	27.8 ± 5.3	29.9 ± 5.8
Age, y	65.6 ± 9.5	67.3 ± 11.5	68.5 ± 9.9
Male^a^	33,159/49,122 (68)	8,383/11,094 (76)	5,894/9,597 (61)
MI^a^	21,521/46,480 (46)	4,428/8,452 (52)	2,583/9,597 (27)
Hypertension^a^	38,394/48,827 (79)	7,064/10,799 (65)	8,591/9,597 (90)
Diabetes mellitus^a^	16,508/48,827 (34)	3,275/10,799 (30)	3,056/9,597 (32)
AF^a^	22,720/44,240 (51)	33,955/9,774 (40)	7,440/8,296 (90)
LVEF, %	34.3 ± 13.0	30.6 ± 9.6	54.3 ± 11.4
NT-proBNP, pg/mL (range)	1,234 (56-19,922)	1,683 (22-28,744)	891 (104-3,651)
Follow-up, mo (range)	37.5 (0.7-112.7)	31.7 (0.2-40.2)	34.0 (1.9-49.8)

Values are n (%), mean ± SD, or n/N (%), unless otherwise indicated.

AF = atrial fibrillation; BMI = body mass index; LVEF = left ventricular ejection fraction: MI = myocardial infarction; NT-proBNP = N-terminal pro-B-type natriuretic peptide; RCT = randomized controlled trial.

a Denominator numbers differ as some data are not available for all cohorts. The total cohort (All) sample size is not the sum of the LVEF strata, as only a subset of cohorts have available LVEF data.

**Table 2:** Heart Failure Clinical Endpoints

	All (N = 50,636)	LVEF ≤40% (n = 12,608)	LVEF >40% (n = 9,597)
Death	11,454 (23)	2,711 (22)	1,029 (11)
CV death or HF hospitalization	11,360 (22)	4,229 (35)	1,600 (17)

Values are n (%).

CV = cardiovascular; HF = heart failure.

Although no genetic variants reached genome-wide significance (*P* < 5 × 10^-8^), 21 independent variants were associated with all-cause mortality and 27 with the composite outcome at a suggestive significance level (*P* < 5 × 10^-6^) (Supplemental Tables 6 and 7). None of the variants with suggestive associations with HF outcomes were associated with new-onset HF in previous GWAS (*P* > 0.01).^8^ We also evaluated the replication of previously reported genetic variants associated with new-onset HF in European ancestry individuals (59 variants) against our GWAS of clinical outcomes in HF. Of these 59 variants, only 4 (11%) were associated with all-cause mortality at a nominal significance of *P* < 0.05, and 7 (20%) were associated with CV mortality or HF hospitalization (35 expected given power difference). The same analysis, but using the 176 genetic variants reported in a multiancestry HF meta-analysis by Lee et al^31^ revealed comparably low replication rates (18% and 16%, respectively) (Supplemental Tables 8 to 11). This limited replication may reflect fundamental differences in the underlying pathobiology of disease onset vs progression or the modifying effects of treatment. To explore potential effect modification by LVEF, we conducted a subgroup GWAS meta-analysis restricted to cohorts with available LVEF data. No additional variants at the genome-wide significance threshold (*P* < 5 × 10^-8^) were identified (Supplemental Tables 12 to 15).

We then applied a range of approaches to identify and correct potential index-event bias influencing the genetic variant associations (Supplemental Methods, Supplemental Figures 1 to 9). To explore the range of bias correction factors under different models of bias adjustment, we conducted sensitivity analyses using multiple bias model and parameter combinations (Supplemental Methods). No genome-wide significant loci were identified after correcting for index-event bias.

### MR study of BMI and clinical outcomes in HF

**Figure 2:**
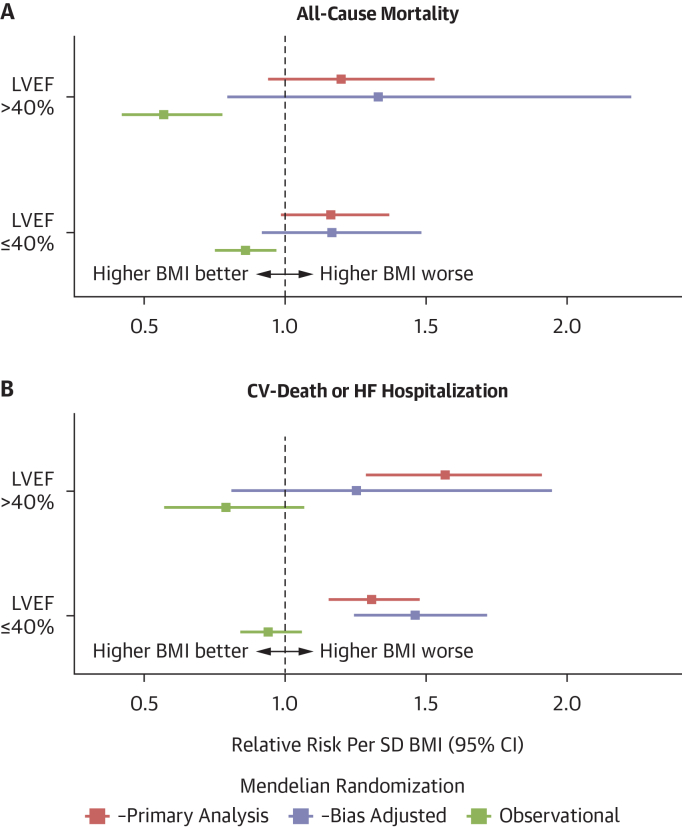
Association of Genetically Predicted BMI with HF Outcomes in Individuals With HFrEF and HFpEF (A) All-cause mortality and (B) cardiovascular death or heart failure hospitalization show the change in HR per standard deviation increase in body mass index in individuals with diagnosed heart failure. Green data points represent previously reported observational associations for HFpEF (Guo et al^52^) and HFrEF (Butt et al^53^) Red data points represent the association derived from the MR analysis of body mass index on HF outcomes. Bars indicate the 95% CI. Purple data points represent the bias-adjusted estimate using the primary bias adjustment method (Bivariate MR CWBLS). HFpEF = heart failure with preserved ejection fraction; HFrEF = heart failure with reduced ejection fraction; other abbreviations as in Figure 1.

Finally, we applied MR to assess the association between genetically predicted BMI and clinical outcomes in individuals with HF. A total of 947 independent variants formed the genetic instrument for BMI (Supplemental Table 4). For the variants included, we did not find evidence of heterogeneity of effects between individuals from the UK Biobank with HF and those without. This confirms that the instrument, derived from a general population, is also valid in patients with HF (*P* > 0.05/947, Bonferroni-adjusted) (Supplemental Figure 10). One SD increment in genetically predicted BMI, corresponding to 4.8 kg/m^2^,^22^ was associated with a 21% higher rate of all-cause mortality (HR: 1.21 per SD; 95% CI: 1.13-1.29; *P* = 9 × 10^-8^). The bias-adjusted CWBLS multivariable MR was consistent with this finding (adjusted HR 1.30 per SD; 95% CI: 1.13-1.48; *P* = 2 × 10^-4^) (Figure 1). There was no evidence of interaction between the association of genetically predicted BMI and all-cause mortality and LVEF subgroup (*P* for interaction 0.84). In subgroup meta-analyses based on study-level LVEF categorization, the association of genetically predicted BMI with all-cause mortality was consistent across LVEF strata. In the LVEF ≤40% subgroup, defined as trials with entry criterion LVEF ≤40% or study mean LVEF ≤35%, the HR was 1.16 per SD BMI (95% CI: 0.92-1.49), whereas in LVEF >40% subgroup—defined as trials with entry criterion LVEF >40% or study mean LVEF >45%—the HR was 1.20 per SD BMI (95% CI: 0.94-1.53) (Figure 2). The bias-adjusted CWBLS multivariable MR results were consistent with these findings (adjusted HR 1.17 per SD; 95% CI: 0.92-1.49 and 1.33 per SD; 95% CI: 0.79-2.23, respectively).

For the composite outcome of CV mortality or HF hospitalization, an SD increment in genetically predicted BMI was associated with a 29% higher rate (bias-adjusted HR 1.29 per SD BMI, 95% CI: 1.20-1.38; *P* = 8 × 10^-13^) (Supplemental Figure 11). The bias-adjusted CWBLS multivariable MR was consistent with this finding (adjusted HR 1.16 per SD; 95% CI: 1.00-1.33; *P* = 0.044). The association was consistent across LVEF strata in subgroup meta-analyses (*P* for interaction 0.13). In the LVEF ≤40% subgroup, each SD increase in BMI was associated with a 31% higher rate of the composite outcome (HR: 1.31 per SD BMI; 95% CI: 1.16-1.48) and in the LVEF >40% subgroup a 57% higher rate (HR: 1.57 per SD BMI; 95% CI: 1.29-1.91) (Figure 2). The bias-adjusted CWBLS multivariable MR results were consistent with these findings (adjusted HR 1.46 per SD; 95% CI: 1.24-1.72 and 1.25 per SD; 95% CI: 0.81-1.95, respectively).

We conducted sensitivity analyses to assess the robustness of these estimates. The weighted median, weighted mode, and MR-Egger regression methods all confirmed the direction of effect for both outcomes, with no evidence of directional horizontal pleiotropy detected (Supplemental Table 16, Supplemental Figures 12 and 13). We then repeated the primary IVW-MR analysis using GWAS outcome data adjusted using different index-event bias correction methods. For all-cause mortality in patients with HF, the bias-adjusted HRs ranged from 1.13 to 1.30, with CIs exceeding the null. For the composite outcome of CV or HF hospitalization, point estimates were all positive (range: 1.03-1.19) but were accompanied by wider CIs, with estimates from 2 methods (Dudbridge and CWLS) overlapping the null (Supplemental Table 16, Supplemental Figure 14). Bias-adjusted estimates in LVEF-stratified analyses were consistently positive but imprecise, with wide CIs that in many cases overlapped the null, reflecting reduced statistical power (Supplemental Table 16, Supplemental Figure 15). The Slope-Hunter method proved highly sensitive to minor variations in input parameters and was deemed unreliable in our dataset (Supplemental Methods, Supplemental Figures 5 to 7). Finally, we repeated the study with a BMI instrument containing no overlapping samples (UK Biobank individuals) and found similar associations with reduced precision (Supplemental Figure 16).

## Discussion

In this study, we investigate the apparent obesity-survival paradox that has influenced clinical thinking about the role of weight loss in the management of patients with HF and obesity. Our observational analysis recapitulated the widely reported finding that higher BMI is associated with lower mortality in patients with established HF. Conversely, our genetic study, which accounts for relevant biases, found that higher BMI is associated with increased mortality in patients with HF. Our index-event bias adjusted estimate of the association of higher BMI on all-cause mortality was similar to the association estimated in the general population using observational analysis. These findings suggest the potential role for weight-loss interventions in the management of HF and provide a rationale for clinical trials.

We did not find evidence of an interaction with LVEF subgroup for the association of BMI on clinical outcomes. However, the magnitude of the effect was numerically greater for HFpEF than HFrEF, consistent with findings from clinical trials of drug-induced weight loss.^14^, ^15^, ^16^ Our results are consistent with a previous 1-sample MR study, which reported a positive association between higher BMI and HF mortality.^40^ Although the effects of specific weight-loss interventions may not be directly comparable with those estimated using genetic proxies, which reflect lifelong exposure to elevated body weight, our findings suggest that weight loss may improve outcomes in HF. Clinical trials of incretin-based therapies are underway in HFpEF, and our findings suggest a rationale for conducting similar trials in patients with HFrEF.

The HF obesity survival paradox was first described >20 years ago, with the observation replicated across numerous studies.^41^^,^^42^ Numerous mechanistic theories have been proposed to explain the paradox, assuming a causal relationship, whereas others suggest that the association is caused by index event bias, a form of collider bias that can arise when studying disease cohorts. Consequently, there persists uncertainty regarding the role of weight reduction in management of patients with HF.^43^, ^44^, ^45^ Selecting populations with HF to study the effects of BMI creates the potential for index-event bias to distort associations between risk factors and their association with prognosis. People with HF and obesity may appear to have a better prognosis not because obesity is causally protective but because they developed HF with fewer or less severe alternative risk factors that have worse effects on prognosis than BMI.^10^ Conventional covariate adjustment cannot mitigate this bias and indeed may amplify it, as we demonstrate in our analysis.^45^^,^^46^

MR describes an approach whereby genetic proxies are used as instrumental variables to model relationships between risk factors and outcomes to mitigate bias. Provided certain assumptions are met, MR can provide estimates that are less susceptible to bias arising from confounding and reverse causation. The associations of genetic variants with prognosis (the outcome), which are necessary for MR, remain vulnerable to index-event bias. However, this bias can be modeled and corrected by using genetic associations with disease onset.^33^^,^^37^ In this study, we apply this by first performing a large time-to-event GWAS in a population with established HF, correct for index event bias, and then performing MR of BMI on clinical outcomes. We demonstrate how this analysis framework can be used to estimate unbiased associations of risk factors on prognosis.

### Study limitations

The GWAS study of clinical outcomes in HF was the largest yet performed; however, larger studies are needed to improve statistical power and to improve the precision of estimates from MR. Furthermore, the study population was limited to individuals of European ancestry because of the limited availability of samples from diverse genetic backgrounds. This is especially relevant, given differing adiposity–cardiovascular risk relationships across populations: for example, higher cardiometabolic risk at lower BMI in South-East Asian individuals, limiting generalizability beyond European ancestry populations.^47^^,^^48^ The subgroup GWAS meta-analysis was limited by the availability of LVEF data in certain studies (only ∼44% of the total cohort, primarily randomized control trials) and by only having access study level LVEF. This will have reduced the power to detect effect modification by LVEF and may affect the external validity of the study. The CIs for the LVEF-stratified estimates were wide and did not consistently exclude the null, indicating limited precision for subgroup-specific causal estimates. Future studies should aim to model the interaction of LVEF as a continuous variable using individual participant-level data.

Our MR analyses, focused on the overall linear association of genetically predicted BMI, acknowledging the J-shaped relationship between BMI and mortality, with underweight individuals (BMI <18.5 kg/m^2^) at higher risk in observational data. Nonlinear MR methods are controversial, and existing methods are insufficient to model this relationship reliably with genetic data.^49^ The small proportion of underweight individuals (for example, <0.5% in the UK Biobank HF population) makes the likely impact on the MR estimates minimal, but if there truly is a causal U-shaped BMI-mortality relationship, the elevated risk at low BMI would be expected to attenuate the MR estimate toward the null (type II error).^50^ BMI is also an imperfect measure of adiposity, as it does not distinguish between fat and lean mass or capture fat distribution, which may influence HF outcomes differentially. In our primary analyses, we used bivariate MR CWBLS to identify and correct the GWAS of HF outcomes for index-event bias. In sensitivity analyses, the majority of alternative bias-adjustment methods produced consistent findings, but the estimates varied, and—in some cases—the CI included the null. Although our MR sensitivity analyses showed consistent direct effects for the primary outcomes, this does not completely exclude pleiotropic bias via mechanisms other than BMI.

## Conclusions

Our findings suggest that obesity is associated with adverse clinical outcomes in HF, similar to the association observed in the general population. They indicate that the obesity survival paradox is likely an artifact of bias, reinforcing the need for caution when making causal interpretations of prognostic associations from observational analyses. Taken together our findings support the potential benefit of weight-loss interventions for patients with HF and may provide a rationale for clinical trials.

### Data Availability

The GWAS summary statistics will be published in full on OpenGWAS, the GWAS catalog (study accessions GCST90827692 and GCST90827693), and the Cardiovascular Knowledge Portal.

The analysis was conducted using a *Snakemake* (version 7.26) workflow.^51^ The code is available in GitHub repositories: https://github.com/nicksunderland/hf_progression_bmi and https://github.com/nicksunderland/hf_progression_bmi_ukbb.

Clinical Perspective **WHAT'S KNOWN?** Observational studies suggest consistently that obesity may be paradoxically protective in patients with heart failure, creating uncertainty about the role of weight loss in clinical management.

**WHAT'S NEW?** Using genetic approaches, this study finds that higher BMI increases the risk of all-cause mortality and cardiovascular events in heart failure, irrespective of ejection fraction, suggesting that the apparent “obesity paradox” is likely caused by bias inherent to conventional observational study designs.

**WHAT'S NEXT?** These findings provide evidence that supports the clinical potential of targeted weight management in care of heart failure. Randomized controlled trials are needed to confirm these findings and to identify specific weight-loss interventions that are safe and effective.

## Funding Support and Author Disclosures

Dr Sunderland is funded by the GW4-CAT HP PhD program. Prof Paternoster and Dr Sunderland work in a Medical Research Council (UKRI)-funded unit (MC_UU_00032/1 and MC_UU_00032/3). This research was supported by the National Institute for Health and Care Research (NIHR) Bristol Biomedical Research Centre (BRC) (NIHR203315). Dr Henry has been supported by the British Heart Foundation Cardiovascular Biomedicine PhD studentship (FS/18/65/34186). Dr Dubé is supported by the Canada Research Chairs Program. Dr Smith has been supported by grants from the Swedish Heart-Lung Foundation (2022-0344, 2023-03332), the Swedish Research Council (2021-02273, 2024-03314), the European Research Council (ERC-STG-2015-679242), Gothenburg University, Skåne University Hospital, governmental funding of clinical research within the Swedish National Health Service, a generous donation from the Knut and Alice Wallenberg foundation to the Wallenberg Center for Molecular Medicine in Lund, and funding from the Swedish Research Council (Strategic Research Area Exodiab Dnr 2009-1039) and Swedish Foundation for Strategic Research (Dnr IRC15-0067) to the Lund University Diabetes Center. Prof Lumbers is supported by the National Institute for Health Research University College London Hospitals Biomedical Research Centre (NIHR203328), National Institutes of Health (5R01HL167509-02), National Institute for Health and Care Research & the Medical Research Council Rare Disease Research UK Cardiovascular Initiative (MR/Y008235/1), and British Heart Foundation Special Project Grant (SP/F/24/150066). The project was additionally supported by a Pfizer Innovative Targets Exploration Network Grant with UCL, the BigData@Heart Consortium, funded by the Innovative Medicines Initiative-2 Joint Undertaking (grant agreement 116074), and the UCL British Heart Foundation Accelerator (AA/18/6/34223). Dr Dubé has received minor equity interest in Dalcor Pharmaceuticals; and has a patent Methods for Treating or Preventing Cardiovascular Disorders and Lowering Risk of Cardiovascular Events issued to Dalcor Pharmaceuticals (no royalties received), a patent Genetic Markers for Predicting Responsiveness to Therapy with HDL-Raising or HDL Mimicking Agent issued to Dalcor Pharmaceuticals (no royalties received), and a patent Methods for using low-dose colchicine after myocardial infarction, assigned to the Montreal Heart Institute. Dr de Denus has been supported through grants from AstraZeneca and Roche Molecular Science/DalCor. Dr O'Donoghue has received grant funding through Brigham and Women’s Hospital from AstraZeneca, Amgen, Novartis, and Marea Therapeutics; and has received consulting and/or DSMB fees from AstraZeneca, Amgen, Janssen, NovoNordisk, Verve Therapeutics, and New Amsterdam. Dr Sabatine has received research grant support through Brigham and Women’s Hospital from Abbott, Amgen, Anthos Therapeutics Inc, AstraZeneca, Boehringer Ingelheim, Daiichi-Sankyo, Ionis, Marea, Merck, Novartis, Pfizer, Saghmos Therapeutics, and Verve Therapeutics; and has received consulting fees from Amgen, AMPEL BioSolutions, Anthos Therapeutics Inc, AstraZeneca, Beren Therapeutics, Boehringer Ingelheim, Dr Reddy’s Laboratories, General Medicines, Merck, Novo Nordisk, and Precision BioSciences. Dr Ruff has received research grants through Anthos, AstraZeneca, Daiichi-Sankyo, Janssen, and Novartis; and has received honoraria for scientific advisory boards and consulting from Anthos, Bayer, Bristol Myers Squibb, Daiichi-Sankyo, Janssen, and Pfizer. Dr White has received grant support paid to the institution and fees for serving on Steering Committees of the ODYSSEY trial from Sanofi and Regeneron Pharmaceuticals, the ISCHAEMIA and MINT study from the National Institutes of Health, the STRENGTH trial from Omthera Pharmaceuticals, the HEART-FID study from American Regent, the DAL-GENE study from DalCor Pharma UK Inc, the AEGIS-II study from CSL Behring, the CLEAR OUTCOMES study from Esperion Therapeutics Inc, the SOLIST-WHF and SCOREDS trials from Sanofi Aventis Australia Pty Ltd, the Librexia and AF and ACS studies from Janssen Research and Development LLC, and the MK0616 Study from Merck Sharp & Dohme Ltd. Dr Hyde was an employee and shareholder of AstraZeneca at the time of the study. Dr Sattar has consulted for and/or received speaker honoraria from Abbott Laboratories, AbbVie, Amgen, AstraZeneca, Boehringer Ingelheim, Carmot Therapeutics, Eli Lilly, GlaxoSmithKline, Hanmi Pharmaceuticals, Menarini-Ricerche, Metsera, Novartis, Novo Nordisk, Pfizer, and Roche; and has received grant support paid to his University from AstraZeneca, Boehringer Ingelheim, Novartis, and Roche outside the submitted work. Dr McMurray has received payments to Glasgow University for clinical trials and other research projects from the British Heart Foundation, National Institute for Health-National Heart, Lung, and Blood Institute (NIH-NHLBI), Alnylam Pharmaceuticals, AstraZeneca, Bayer, Cardurion, Cytokinetics, Novartis, and Roche; has received personal consultancy fees from Alnylam Pharmaceuticals, AnaCardio, AstraZeneca, Bayer, Cardurion, Cytokinetics, Novartis, River BioMedics, Biohaven Pharmaceuticals, Chugai Pharmaceuticals, Protherics Medicine Developments Ltd, and DalCor Pharmaceuticals; and has received personal lecture fees from Alkem Metabolics, AstraZeneca, Canadian Medical and Surgical Knowledge, Centrix Healthcare, Emcure Pharmaceuticals, Eris Lifesciences, Hikma Pharmaceuticals, Imagica Health, Intas Pharmaceuticals, J.B. Chemicals & Pharmaceuticals, Lupin Pharmaceuticals, Medscape/Heart.Org, ProAdWise Communications, Radcliffe Cardiology, Sun Pharmaceuticals, Translational Medicine Academy, Regeneron, MCI India, Hilton Pharmaceuticals, IMEDIC Pharmaceuticals Micro Labs Ltd, At the Limits Ltd, and ARMGO Pharmaceuticals; has served on Data Safety Monitoring Boards for WCG Clinical Services; and is director of Global Clinical Trial Partners Ltd (which provides clinical trial services such as endpoint committees and educational programs). All other authors have reported that they have no relationships relevant to the contents of this paper to disclose.
