# Supplementary material for: Body Mass Index, Clinical Outcomes, and Mortality in Heart Failure: A Mendelian Randomization Study
Source: J Am Coll Cardiol. 2026 Jun 2;87(21):2981–92. doi: 10.1016/j.jacc.2026.02.5093 (PMC13211948; doi:10.1016/j.jacc.2026.02.5093)
Supplement: Supplemental Material 1 [file mmc1.docx]

**Supplement**

**Index-event bias sensitivity analyses**

Genome-wide association studies (GWAS) of disease severity, prognosis, or survival are vulnerable to index event bias.[^1^](https://www.zotero.org/google-docs/?NUehuh) This is relevant to our analyses, where we conducted a time-to-event GWAS restricted to HF patients, examining endpoints of all-cause death, and the composite outcome of cardiovascular death or heart failure hospitalization. Selecting only HF cases to study progression can induce correlation between independent HF causes, genetic and nongenetic, and these outcomes (**Figure 1**).


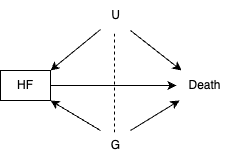


**Figure 1.** Directed acyclic graph of association of SNP **G** with outcome death conditional on heart failure (HF). U is a composite variable including all common causes of HF and death, and may include polygenic effects as well as non-genetic risk factors. Conditioning on HF induces an association between G and U (dashed line), and creates an association between G and death via the path G - U → death in addition to the direct effect of G → death.

A key question in case-only GWAS like ours is the extent to which genetic associations with progression traits (death or the composite outcome) are distorted by index event bias. Most adjustment methods use GWAS summary statistics, although inverse probability weighting methods can be used when individual level data are available.[^2^](https://www.zotero.org/google-docs/?rJ9dFd) In the following discussion we describe these summary methods in relation to our heart failure datasets.

Methods to assess this bias generally assume that unmeasured confounder (U) effects are approximately constant across SNPs (G). Under this, the bias can be estimated by regressing SNP effects on disease progression (from our meta-analysis) on SNP effects on disease incidence (from a case-control GWAS of HF onset).[^1^](https://www.zotero.org/google-docs/?AxTEnU) For statistical validity, this regression requires independent SNPs, so genome-wide variants must first be pruned via linkage disequilibrium (LD) clumping. Genome-wide variants are therefore pruned using PLINK 2.0 (--indep-pairwise) at a 250 kb window size and r^2^ threshold of 0.1.[^1,3,4^](https://www.zotero.org/google-docs/?fafmuA)

The general procedure estimates bias due to selection by regressing the independent genetic effects (or some subset, described later) on HF progression, against the corresponding effects on HF incidence (**Figure 2**). The slope of the relationship provides an estimate of the bias attributable to index-event bias. Various methods implement this differently:

- **Dudbridge et al. 2019**: uses all genome-wide variants in a standard regression with an intercept.[^1^](https://www.zotero.org/google-docs/?86dFWo)
- **Corrected weighted least squares (CWLS)**: uses all genome-wide variants in a zero-intercept regression, corrected for weak instruments.[^3^](https://www.zotero.org/google-docs/?HPyuZZ)
- **Slope-Hunter**: identifies ‘valid’ instrumental variables (IVs) through a model-based clustering algorithm, and performs the regression on this subset.[^5^](https://www.zotero.org/google-docs/?Fwmie9)
- **Mendelian randomisation methods**: applies standard MR methods (e.g. inverse variance weighted (IVW), weighted-median, or weighted-mode, MR-Egger) to genome-wide significant SNPs for HF onset (eg, *P* value < 5 × 10^-8^).[^2^](https://www.zotero.org/google-docs/?KUZ0ov)


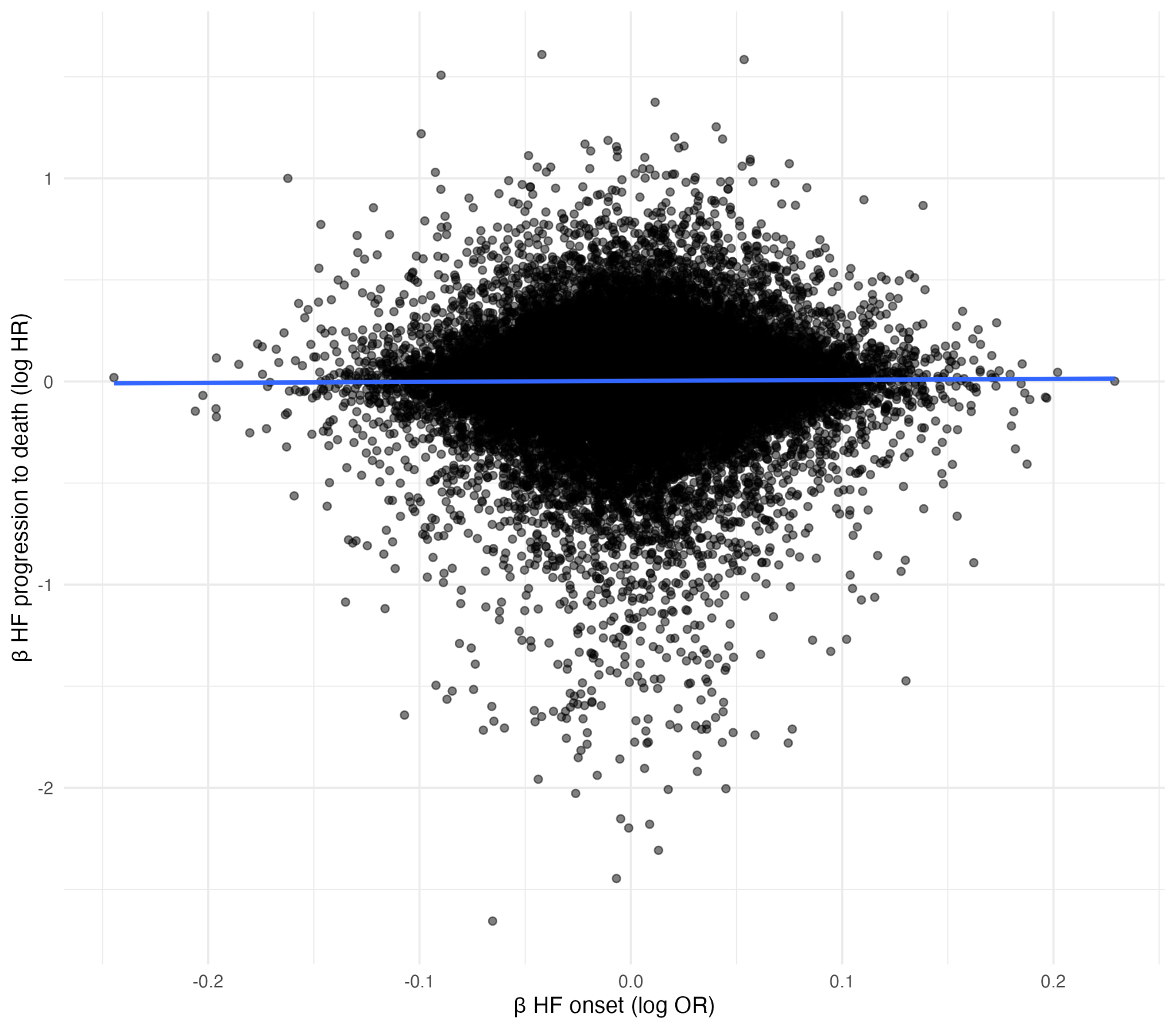


**Figure 2.** One example of the genetic associations with all-cause heart failure (HF) onset (x-axis) versus HF progression to death (y-axis) for 267,941 genome-wide independent variants linkage disequilibrium pruned (*r2* = 0.1, *kb* = 250). Methods to assess index event bias aim to estimate the slope of the regression line through these points (or a subset of points), which represents the bias arising from selection. The blue line presents an unweighted regression *lm(progression_beta ~ incidence_beta)*, however the various bias methods implement this regression slightly differently.

**Dudbridge et al. 2019**

The Dudbridge et al. method assumes the observed SNP-progression association is the true progression effect plus a bias linear in the SNP’s incident (HF onset) effect.[^1,2^](https://www.zotero.org/google-docs/?D97pvj) For our death endpoint:

$$\beta_{death}^{est} =\beta_{death}^{true} +b\beta_{HF}^{true}$$

Where $\beta_{death}^{est}$is the observed SNP effect estimate from the time-to-death GWAS of individuals with diagnosed HF, $\beta_{HF}^{true}$ is the SNP effect on HF incidence (case-control GWAS), $\beta_{death}^{true}$ is the true effect of the SNP on HF progression to death (effect of interest), and *b* is the slope from a regression of $\beta_{death}^{est}$ on $\beta_{HF}^{true}$ (the bias correction factor).

It uses all LD-pruned genome-wide SNPs to estimate *b*, the correction factor, assuming linear effects without interaction (no correlation between a SNPs effect on incidence and its direct effect on progression). However, this is debatable, particularly for cardiovascular diseases such as HF, where shared pathways plausibly link incidence and progression.[^2^](https://www.zotero.org/google-docs/?Tx8gZz) The heritability and genetic correlation of our progression and incidence traits was assessed using LD score regression.[^6^](https://www.zotero.org/google-docs/?Q7PDWB) Table 1 highlights that the HF progression traits have very low heritability (*h^2^*), for example in all-cause HF *h*²=0.012 (95% CI: –0.009–0.033, *P* = 0.275) for the GWAS of all-cause mortality and *h*²=0.004 (95% CI: –0.012–0.020, *P* = 0.624) for the composite endpoint. Such low heritability means that estimates of genetic correlation with incidence are inherently noisy.

|  | **All-cause heart failure** | **Heart failure preserved LVEF** | **Heart failure reduced LVEF** |
| --- | --- | --- | --- |
| Incidence (h^2^ liability) | 0.026 (0.023-0.029; p = 0.000) | 0.056 (0.047 0.064; p = 0.000) | 0.056 (0.049-0.063; p = 0.000) |
| Progression (h^2^ observed)  All cause mortality | 0.012 (-0.009-0.033; p = 0.275) | 0.028 (–0.081 to 0.137; p = 0.613) | 0.053 (–0.034-0.141; p = 0.234) |
| Progression (h^2^ observed)  Composite 1 | 0.004 (-0.012-0.020; p = 0.624) | 0.027 (–0.073 to 0.127; p = 0.592) | 0.006 (–0.081-0.092; p = 0.900) |

**Table 1.** SNP-based heritability estimates for heart failure incidence (on the liability scale) and progression traits (on the observed scale). Values are presented as h^2^ with 95% confidence intervals and *p*-values.

As shown in Table 2, while we observed high and sometimes >1 estimates for genetic correlation (eg, *r*_g_ = 1.50, 95% CI: 1.03-1.97), as the LD score regression method is unbounded, these almost certainly reflect statistical noise. The overall interpretation is that incidence and progression GWAS may share some genetic signal, but that the estimates are dominated by noise owing to lack of genetic heritability.

|  | **Incident**  **all-cause heart failure** | **Incident**  **heart failure preserved LVEF** | **Incident**  **heart failure reduced LVEF** |
| --- | --- | --- | --- |
| **Progression to**  **all-cause mortality** | 0.485 (0.192-0.777; p = 0.001) | 0.252 (–0.342 to 0.846; p = 0.405) | 0.272 (–0.041 to 0.584; p = 0.089) |
| **Progression to**  **composite 1** | 1.501 (1.028-1.974; p = 0.000) | 0.032 (–0.488 to 0.551; p = 0.905) | 0.999 (0.088-1.910; p = 0.032) |

**Table 2.** Genetic correlations r_g_ between heart failure incidence subtypes (all-cause, preserved LVEF, and reduced LVEF) and progression traits (all-cause mortality and a composite endpoint of cardiovascular death or heart failure hospitalization). Values are presented as r_g_ with 95% CIs and *P* values.

A further assumption is constant confounding across SNPs (genetic and non-genetic confounding), which could fail with significant genetic correlation between incidence and progression, as the genetic component of the unmeasured confounding will be weaker for SNPs that are strongly associated with both incidence and progression.[^2^](https://www.zotero.org/google-docs/?SKlsrP) Despite this, simulations show reduced type 1 error versus unadjusted analysis, even where positive correlation exists.[^1^](https://www.zotero.org/google-docs/?5MeUUb) Simulations have shown that the only situation where the Dudbridge method perform worse than an unadjusted analysis is under strong negative (*r*_g_ = –0.45) genetic correlation.[^1^](https://www.zotero.org/google-docs/?4RKgAJ)

Regression dilution, where error in the SNP-incidence associations biases the estimated slope towards the null, is addressed in the Dudbridge method via two adjustment options: the Hedges-Olkin estimator or a simulation extrapolation (SIMEX) approach. The SIMEX approach is recommended in real datasets.[^1^](https://www.zotero.org/google-docs/?rv4Zz8)

Dudbridge et al. note that the regression should be performed in well imputed SNPs.[^1^](https://www.zotero.org/google-docs/?9eg1se) In our meta-analyses, we applied an imputation INFO score threshold of 0.5 and restricted inclusion to variants with minor allele frequency (MAF) >1%. However, we observed that low frequency variants can yield unstable and sometimes implausible effect estimates (eg, hazard ratios > 100), likely due to the small number of events carried on rare alleles. To investigate the impact of this on bias assessment we performed analyses at increasing MAF thresholds. Table 3 presents the number of independent variants available for analysis after harmonisation of the incidence and progression GWAS datasets, using the pruning methodology described above, and at different MAF thresholds.

|  | **All-cause heart failure** | | | |
| --- | --- | --- | --- | --- |
| **Outcome** | **All-cause mortality** | | **CV death or HF hospitalisation** | |
| ***MAF threshold*** | **No. SNPs** | **Covariance** | **No. SNPs** | **Covariance** |
| 0.00 | 267,978 | 2.25E-05 | 273,930 | 1.66E-05 |
| 0.01 | 267,978 | 2.25E-05 | 273,930 | 1.66E-05 |
| 0.02 | 162,252 | 1.56E-06 | 162,395 | 1.11E-05 |
| 0.03 | 125,869 | 3.48E-07 | 125,857 | 5.95E-06 |
| 0.04 | 105,181 | -9.03E-08 | 105,021 | 3.75E-06 |
| 0.05 | 91,521 | 4.46E-07 | 91,315 | 3.66E-06 |
| 0.06 | 81,367 | 6.62E-07 | 81,257 | 3.96E-06 |
| 0.07 | 73,571 | 3.52E-07 | 73,546 | 3.24E-06 |
| 0.08 | 67,758 | 2.43E-07 | 67,762 | 3.21E-06 |
| 0.09 | 63,456 | 2.15E-07 | 63,457 | 2.82E-06 |
| 0.10 | 60,360 | 6.62E-08 | 60,368 | 2.12E-06 |

**Table 3.** number of variants (No. SNPs) available for analysis after harmonisation of the incidence and progression datasets, pruned for independence using r^2^ 0.1 and window 250kb, and at different minor allele frequency (MAF) thresholds. Additionally, the covariance between incidence and progression SNP effects are presented.

The bias correction factor estimates for the Dudbridge et al. (2019) method using genome-wide independent SNPs at different minor allele frequency (MAF) inclusion thresholds are presented in **Figure 3**. The correction factor is influenced by the inclusion of unstable estimates from low-frequency variants but stabilizes when common variants (MAF >5%) are used. This behaviour can be explained by their adjustments for regression dilution - low-frequency variants have larger standard errors, which inflate the measurement-error term in the denominator of the regression dilution correction formula, leading to an exaggerated slope. Removing these variants reduces the mean squared SE contribution, increases the effective denominator, and produces a more stable bias correction factor.[^1^](https://www.zotero.org/google-docs/?Bprer2) At a MAF inclusion threshold of >5%, the bias correction factor estimates for all-cause HF and the progression traits of all-cause mortality and the composite outcome were: 0.127 (95% CI: 0.125-0.128) and 0.456 (95% CI: 0.455-0.458), respectively.


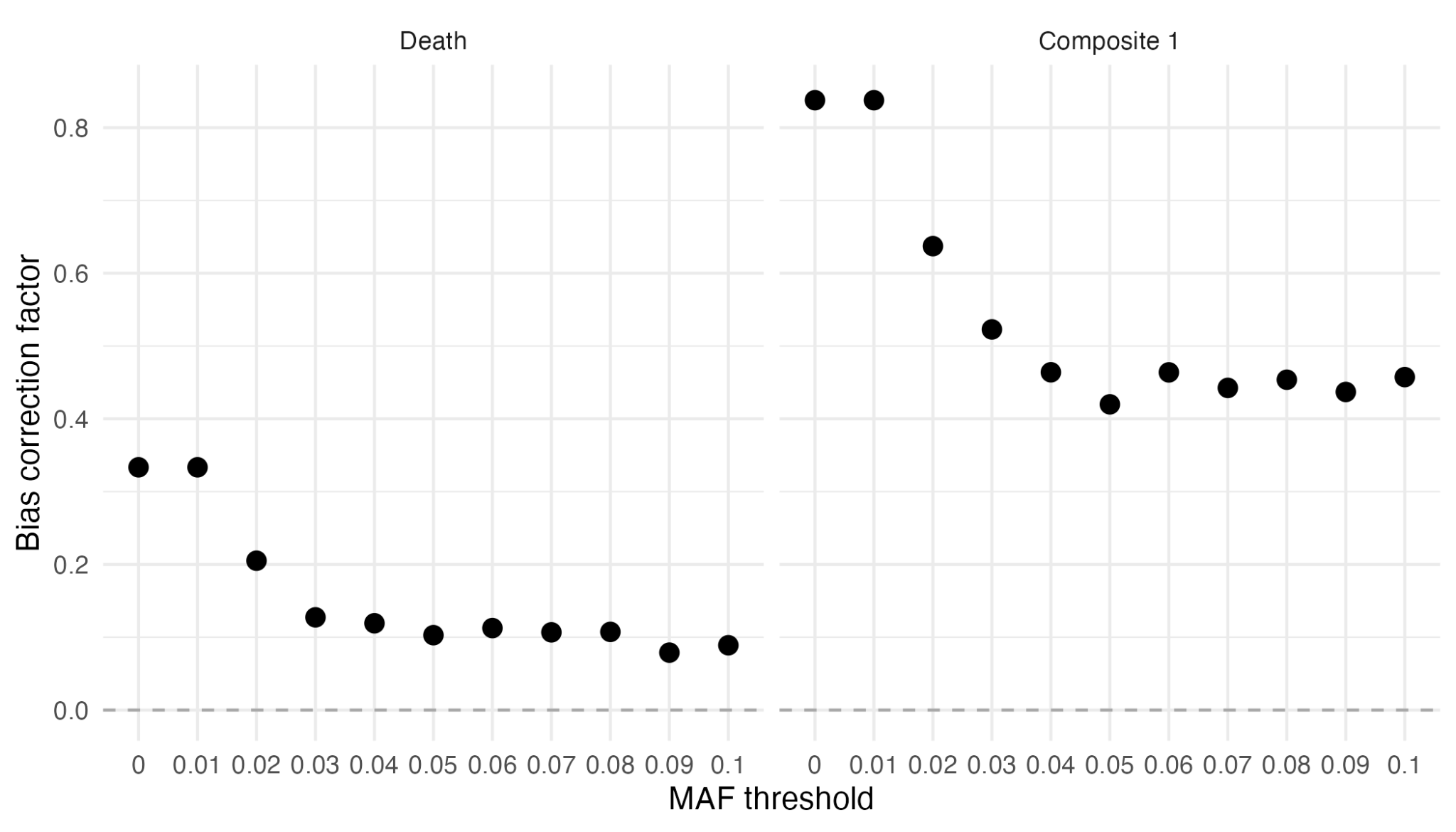
**Figure 3.** Bias correction factors for the all-cause mortality and composite endpoint progression traits in all-cause heart failure, obtained using the Dudbridge method (R package *indexevent*), pruned genome-wide SNPs (*r2* = 0.1, *kb* = 250) with varying MAF inclusion threshold, and the recommended SIMEX adjustment for regression dilution.

**Corrected weighted least squares (CWLS)**

Cai et al. provided an updated version of the Dudbridge method, recommending a non-intercept model called Corrected weighted least squares (CWLS).[^3^](https://www.zotero.org/google-docs/?4WVLPO) The model still assumes no genetic correlation between incidence and progression, but demonstrated a lower type 1 error rate compared to the original method provided there is no strong negative genetic correlation between the incidence and progression trait.[^3^](https://www.zotero.org/google-docs/?uUFi6g)

The bias correction factor estimates for the CWLS method using genome-wide independent SNPs at different minor allele frequency (MAF) inclusion thresholds are presented in **Figure 4**. The correction factor is again influenced by the inclusion of unstable estimates from low-frequency variants but stabilizes when common variants (MAF >5%) are used. At a MAF inclusion threshold of >5%, the bias correction factor estimates for all-cause HF and the progression traits of all-cause mortality and the composite outcome were: 0.129

(95% CI: –0.002 to 0.259 and 0.443 (95% CI: 0.311-0.574), respectively.


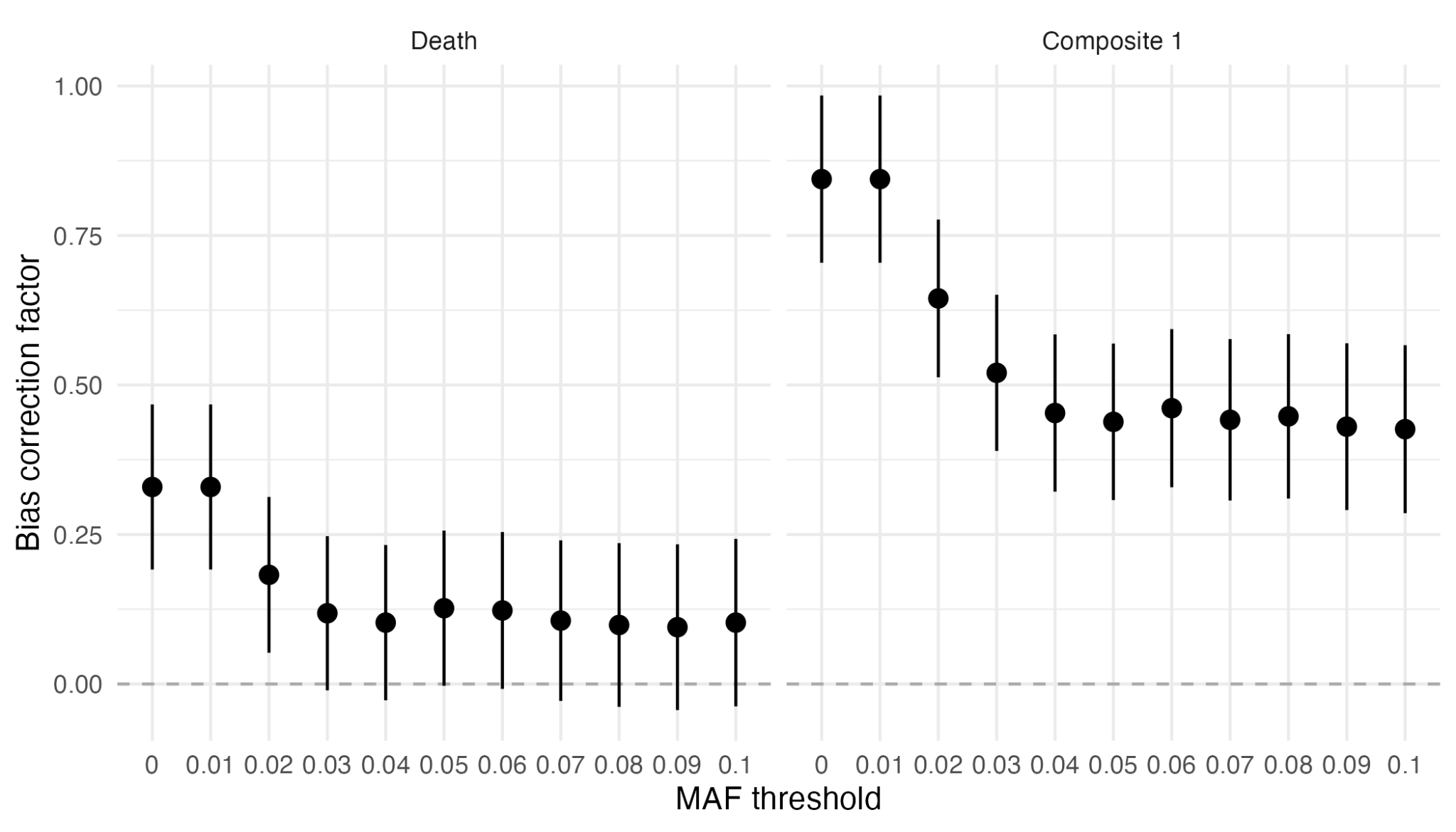
**Figure 4.** Bias correction factors for the all-cause mortality and composite endpoint progression traits in all-cause heart failure, obtained using the updated CWLS Dudbridge method (R package *indexevent*), pruned genome-wide SNPs (*r^2^* = 0.1, *kb* = 250) with varying MAF inclusion threshold.

**Slope-Hunter**

The Slope-Hunter method was developed to try to overcome the assumption of no genetic correlation between incidence and progression.[^5^](https://www.zotero.org/google-docs/?PRnRmU) The method aims to first identify independent SNPs that are associated with disease incidence (HF onset). SNPs that do not associate with disease incidence, that is, only associate with disease progression (G_P_), or associate with neither (G_.._), will not suffer from index-event bias when conditioning on HF (lack of G → HF association in **Figure 1**). These SNPs (G_P_ and G_.._) are first filtered out using a P-value threshold on the incidence association ($\lambda$, default < 0.001). Taking putative disease incidence SNPs, the method then uses an expectation-maximisation (EM) algorithm to cluster the SNPs into 1) those that only affect incidence (G_I_), and 2) those affecting both incidence and progression (G_IP_).

In the EM algorithm, each cluster is modelled as a bivariate Gaussian within a covariance structure. For the cluster representing SNPs only affecting incidence (G_I_), the covariance is initially set to be strongly positive (or negative) along a diagonal, in accordance with the covariance of the raw input data. The method enforces the sign (–1 or +1) of this covariance even if the raw value is near zero. During the E-step, SNPs are assigned posterior probabilities of belonging to the strongly oriented cluster based on how closely they lie along the artificial diagonal, and during the M-step, the cluster parameters are updated using these weighted assignments. This iterative process selectively upweights SNPs that happen to fall along the initial diagonal orientation, thereby reinforcing the apparent slope. As a result, even when the underlying genetic correlation is near zero, the EM procedure can produce a regression slope that appears strongly positive (or negative) because the algorithm effectively anchors the cluster along the prespecified covariance direction.

The final regression is then performed within the G_I_ SNPs, those identified as only affecting disease onset and in which any association with progression is due to bias - the correction factor (*b*_1_) is then the slope of this regression line.


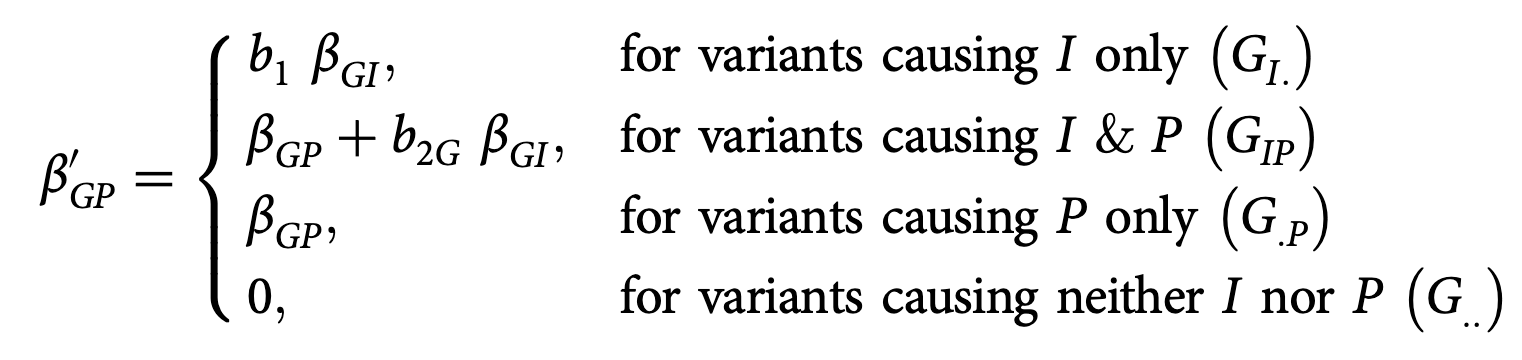


As described, non-incidence SNPs (G_P_ and G_.._) are removed using a P-value threshold ($\lambda$) for the SNP association with disease incidence. The suggested default is $\lambda$ = 0.001 for exclusion, although exploring a range of P-value thresholds is recommended.[^2,5^](https://www.zotero.org/google-docs/?k5rP6Z)

We explored different $\lambda$ thresholds around the suggested default of 0.001. The bias correction factor estimate obtained by the Slope-Hunter method is unstable in our data, ranging from very positive to very negative values (**Figure 5**). We see that the model amplifies the covariance structure of the input SNPs, despite this being essentially zero. Small changes in the choice of $\lambda$ can lead to subtle fluctuations in the input covariance around zero resulting in drastically different bias correction factors. For example $\lambda$ = 0.001 (covariance -8.5 x 10^-5^) yields a correction factor of –0.33 (95% CI: –0.52 to –0.15) (**Figure 6**), but only a small increase in $\lambda$ to 0.0011 (covariance +6.4 × 10^-5^) yields a large positive correction factor of 0.42 (95% CI 0.24-0.61) (**Figure 7**). Attempts to stabilize the estimates by filtering on increasing MAF (eg, >5%) lead to failure of the model to assign any G_I_ SNPs at the recommended $\lambda$ = 0.001. Wide variation and conflicting bias estimates have also been reported using the Slope-Hunter method in other datasets.[^7^](https://www.zotero.org/google-docs/?cm2Ud9) For these reasons we consider the method unreliable in our data.


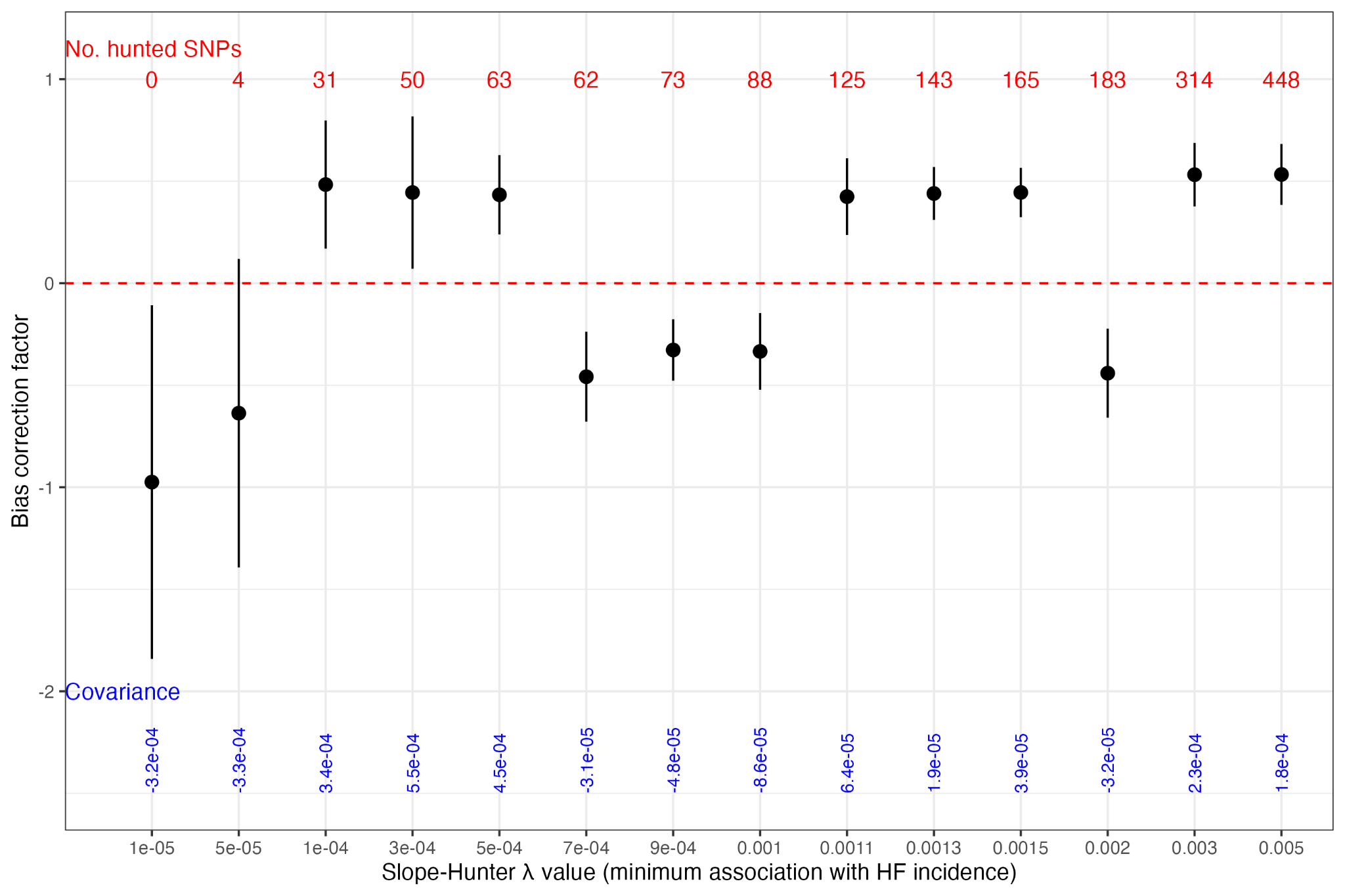
**Figure 5.** Bias correction factors (y-axis) for the all-cause mortality endpoint progression trait in all-cause heart failure, obtained using the Slope-Hunter method (R package *Slopehunter*), pruned genome-wide SNPs (*r^2^* = 0.1, *kb* = 250), MAF > 1%, and varying the P-value threshold $\lambda$ (x-axis) for inclusion of putative incidence SNPs. The number of ‘hunted’ SNPs (G_I_) is shown in red, and the covariance between the input incidence and progression SNPs is shown in blue. The covariance is close to zero for all $\lambda$ values, whoever where the covariance has a negative sign, the bias estimate is negative, and when it has a positive sign the bias estimate is positive.


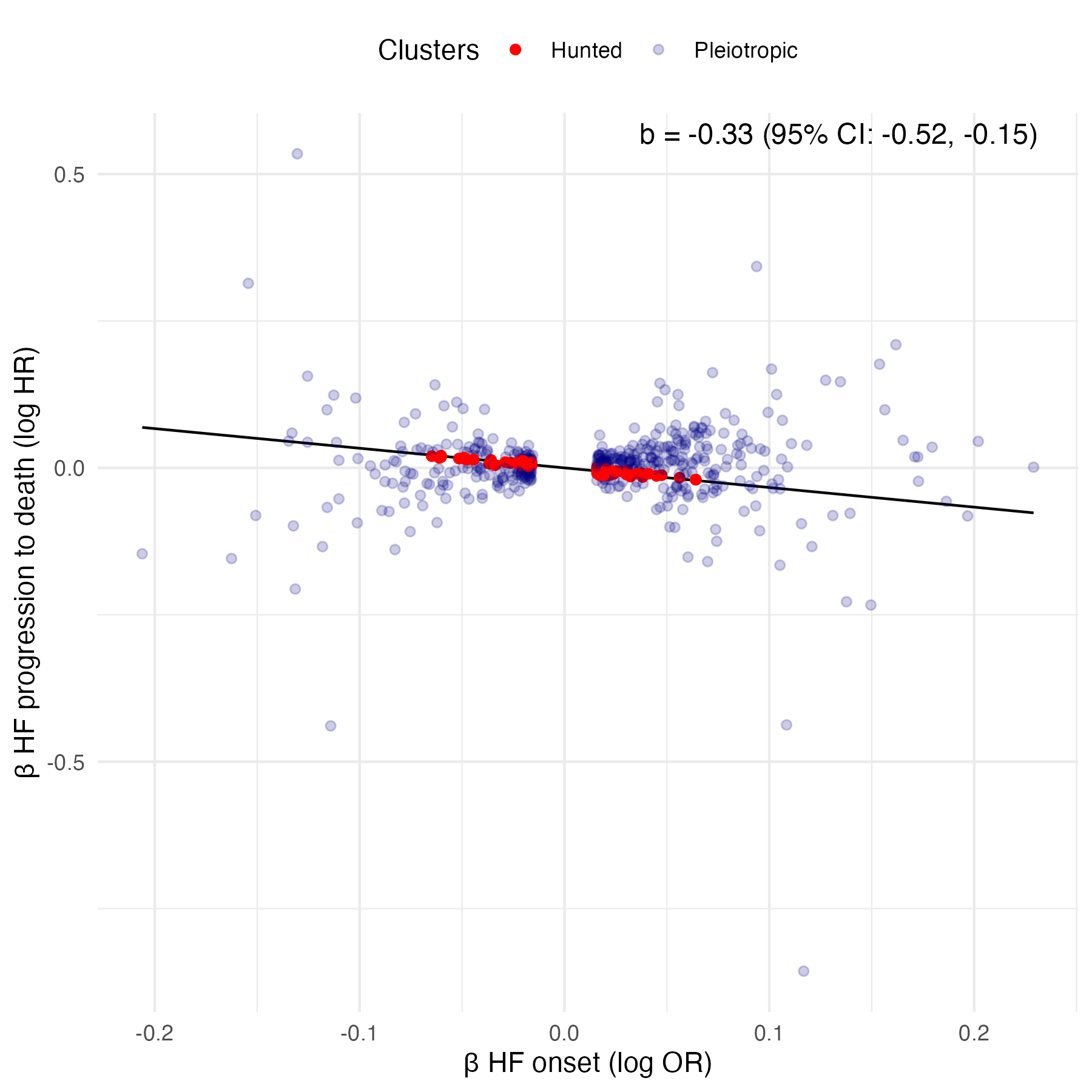


**Figure 6.** An example of the Slope-Hunter method using genome-wide independent variants prune with linkage disequilibrium parameters (*r2* = 0.1, *kb* = 250) and method parameters ip ($\lambda$) = 0.001, pi0 ($\pi$) = 0.6, sxy1 ( $\hat{\sigma}_{IP}$) = 1 × 10^-5^. Genetic associations with heart failure (HF) onset (x-axis) vs HF progression to death (y-axis). The Slope-Hunter method assigns 88 SNPs (red) to the "incidence-only effect" group 1. The regression slope through these points yields the bias correction factor -0.33 (95% CI: –0.52 to –0.15).


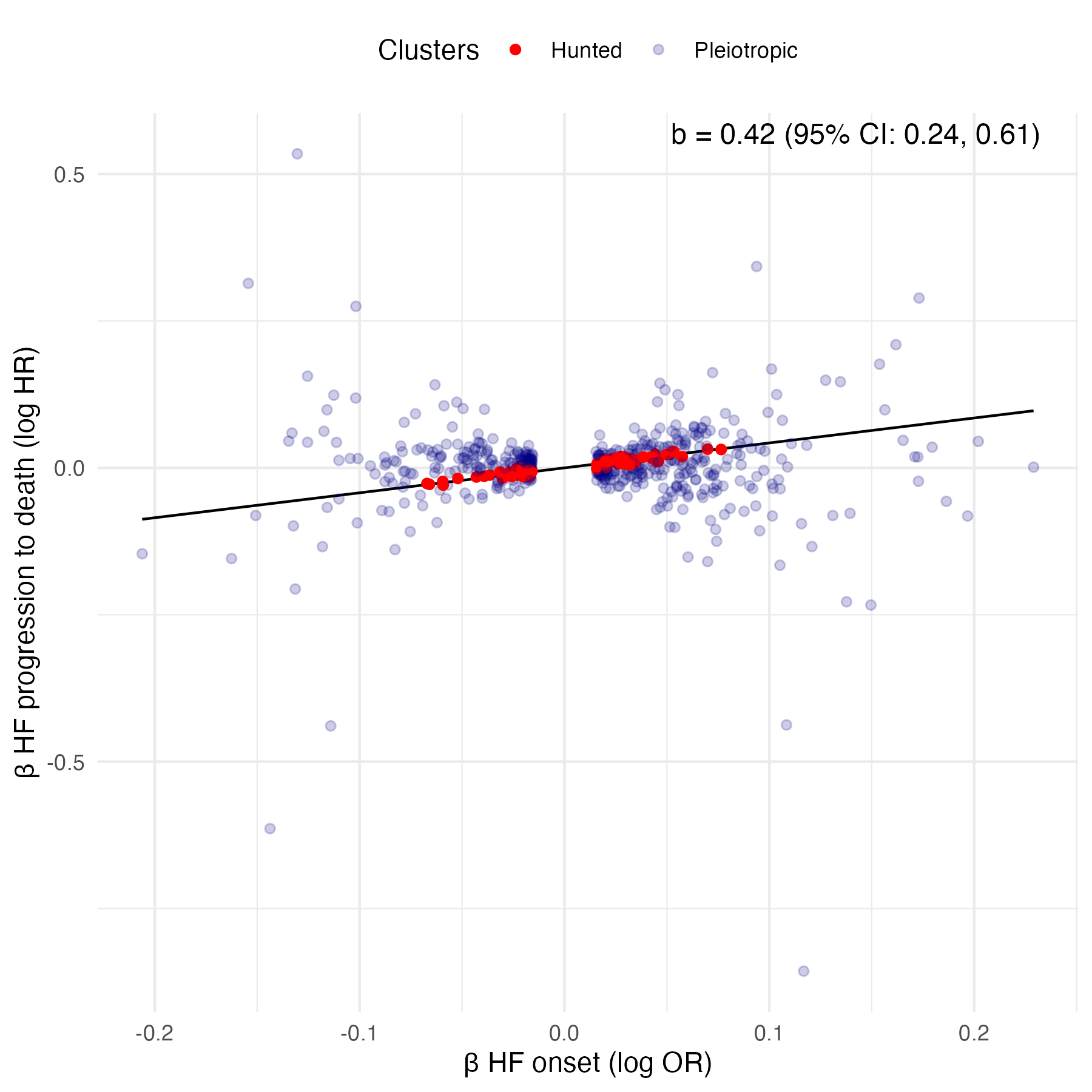


**Figure 7.** An example of the Slope-Hunter method using genome-wide independent variants prune with linkage disequilibrium parameters (*r2* = 0.1, *kb* = 250) and method parameters ip ($\lambda$) = 0.0011, pi0 ($\pi$) = 0.6, sxy1 ( $\hat{\sigma}_{IP}$) = 1x10^-5^. Genetic associations with heart failure (HF) onset (x-axis) versus HF progression to death (y-axis). The Slope-Hunter method assigns 88 SNPs (red) to the ‘incidence-only effect’ group 1. The regression slope through these points yields the bias correction factor +0.42 (95% CI 0.24-0.61).

**Mendelian randomization methods**

Two-sample MR approaches have been suggested as a way to estimate the slope between SNP effects on incidence and their corresponding effects on progression - the bias correction factor.[^2,3^](https://www.zotero.org/google-docs/?QIhhsM) The main contrast with standard two-sample MR is that the analysis can be conducted within a single sample (sample overlap).[^3^](https://www.zotero.org/google-docs/?Rmjv55)

In two-sample MR, weak instruments - where SNPs explain only a small proportion of the variance in the exposure - tend to bias the MR estimate towards the null. In the context of index event bias this may lead to underestimation of the slope and consequent underadjustment of the progression GWAS effect estimates. In standard MR applications, instrument SNPs are typically selected based on strong association with the exposure, something that also has been recommended in the setting of assessing index event bias.[^2^](https://www.zotero.org/google-docs/?LozI4z) However, Cai et al. argue that if a limited number of SNPs are then available as instrumental variables, such selection may undermine assumptions needed for slope estimation.[^3^](https://www.zotero.org/google-docs/?ZfxHKa) Specifically, the Dudbridge et al. and the updated CWLS method rely on assumptions about the distribution of direct outcome effects across many SNPs, including the InCLUDE (instrument coefficient linearly uncorrelated with direct effect) assumption, analogous to the InSIDE assumption in MR-Egger. With only a handful of strong instruments, it may be difficult to justify these assumptions, as their direct effects on the outcome (HF progression) may reflect specific biological pathways and thus be correlated with effects on HF onset. In contrast, when a very large number of genome-wide SNPs are included, they argue that collectively the assumption becomes more plausible. They therefore recommended using a large set of SNPs.[^3^](https://www.zotero.org/google-docs/?CTtGas) Nevertheless, we include the standard MR analyses as a recommended sensitivity analysis (**Figure 8**).[^2^](https://www.zotero.org/google-docs/?twqCh5)


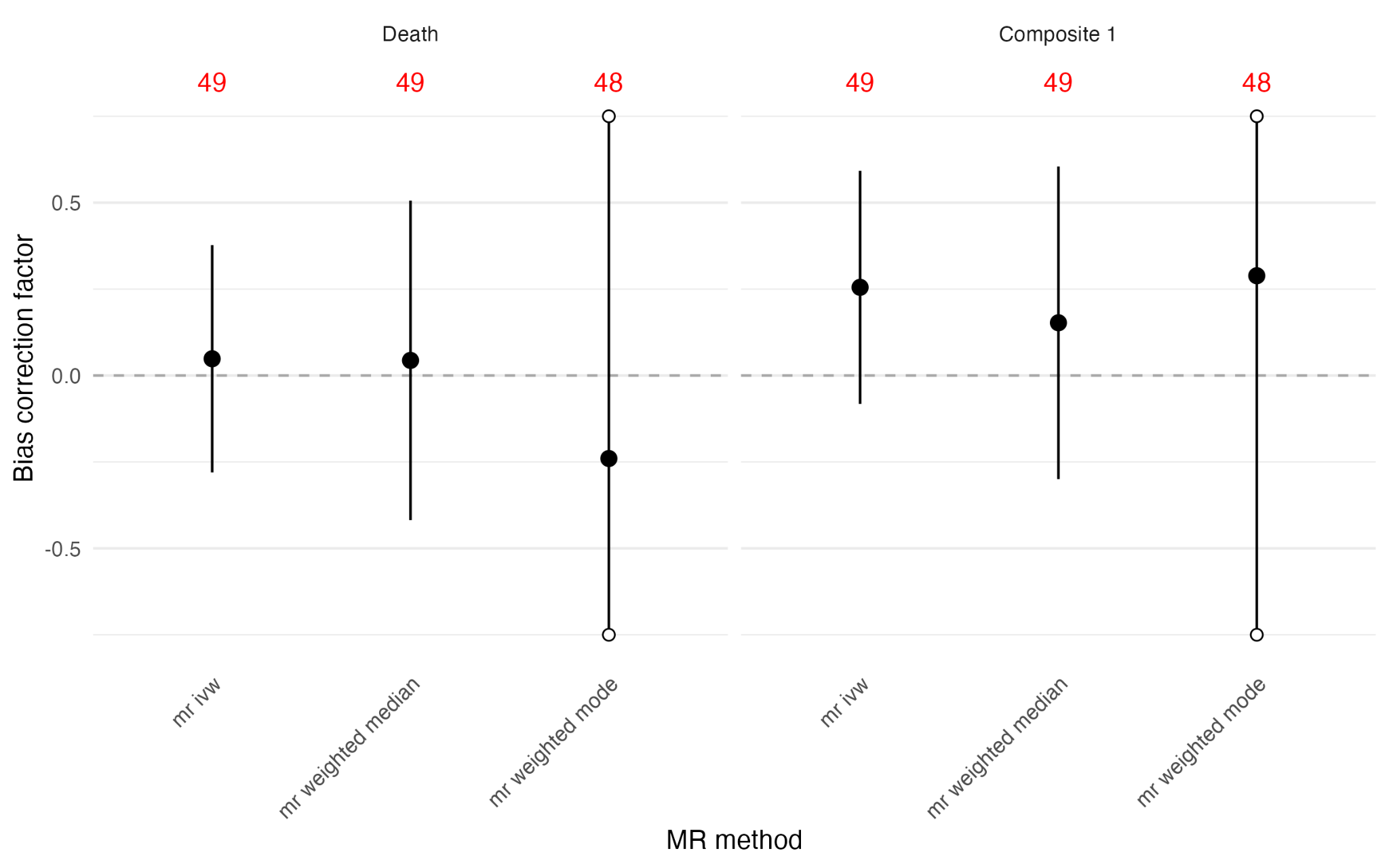
**Figure 8.** Bias correction factors (y-axis) for the all-cause mortality and composite endpoint progression traits in all-cause heart failure, obtained using standard Mendelian randomization methods (x-axis) (R package *MendelianRandomization*), and clumped genome-wide incidence SNPs (*r2* = 0.001, *kb* = 10,000, p1 = 5e-8) with MAF > 1%. The number of SNPs in the instrument is shown in red. The error bars represent 95% confidence intervals and open circles clipped data.

**Application of the bias correction factor**

Once the bias correction factor has been obtained, it can be used to adjust the HF progression GWAS data using the formulae:

[
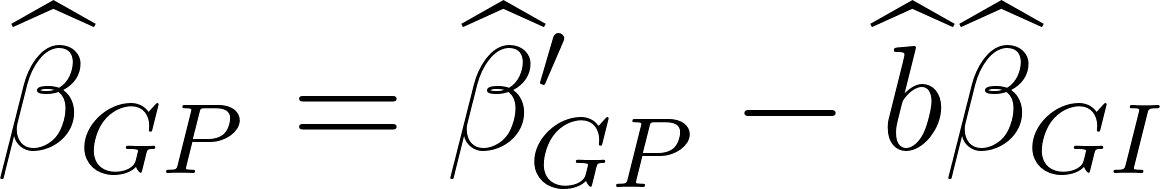
](https://www.codecogs.com/eqnedit.php?latex=%5Cwidehat%5Cbeta_%7BGP%7D%20%3D%20%5Cwidehat%5Cbeta%5E%5Cprime_%7BGP%7D%20-%20%5Cwidehat%7Bb%7D%20%5Cwidehat%5Cbeta_%7BGI%7D#0)

[
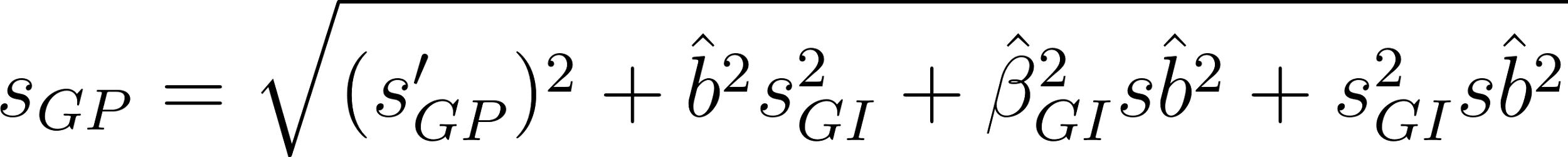
](https://www.codecogs.com/eqnedit.php?latex=s_%7BGP%7D%20%3D%20%5Csqrt%7B%20(s%5E%5Cprime_%7BGP%7D)%5E2%20%2B%20%5Chat%7Bb%7D%5E2%20s_%7BGI%7D%5E2%20%2B%20%5Chat%5Cbeta_%7BGI%7D%5E2%20s%20%5Chat%7Bb%7D%5E2%20%2B%20s_%7BGI%7D%5E2s%20%5Chat%7Bb%7D%5E2%20%7D#0)

Where $\beta_{GP}$ is the true association of a SNP with HF progression to death, or the composite outcome; $\beta_{GP}^{'}$ is the biased association with HF progression from our GWAS meta-analysis; $\beta_{GI}$ is the association with HF incidence (onset); *b* bias correction factor; $s_{GP}$ the true standard error of the association with HF progression; $s_{GP}^{'}$ the biased standard error of the association with HF progression from our GWAS meta-analysis; and $s_{GI}$ the standard error of the association with HF incidence.

Once the progression GWAS has been adjusted, the MR analysis of BMI on HF progression is then repeated with this updated outcome data.

**Bivariate Mendelian Randomisation**

A recently proposed method to account for index-event bias when estimating the causal effect of an exposure on a progression trait is bivariate Mendelian randomisation, in combination with a form of CWLS adjustment.[^8^](https://www.zotero.org/google-docs/?OCsE5m) This method is a form of multivariable MR, regarding disease progression as the outcome and considering two exposures: the exposure of interest (BMI in our case) and disease incidence (HF onset). As for the original Dudbridge and CWLS methods, a large number of SNPs are recommended when correcting for index event bias using instrument effect regression (in our case: 84,187 for all-cause death, and 84,006 for the composite endpoint of CV-death or HF hospitalisation), and therefore correction for weak instruments is necessary. Dudbridge et al. have extended the CWLS estimator to a bivariate form (CWBLS) to adjust for weak instruments in this setting.[^8^](https://www.zotero.org/google-docs/?ZHIrTU)

A key development is the relaxation of the assumption of causal independence of exposure and disease traits, in our scenario allowing causal effects between BMI, HF incidence, and HF progression traits. The method assumes that measurement error in the marginal effects of exposure (BMI) and disease (HF onset) traits are independent, plausible if BMI estimates are drawn from separate samples. In this work we show that the small overlap of UK Biobank samples between the GWAS for BMI and our HF traits has limited impact (**Figure 15**). In addition, the instrument coefficient linearly uncorrelated with direct effect (InCLUDE) assumption is required, i.e. the direct genetic effects on disease progression are uncorrelated with the effects on disease incidence and on the exposure. Cai and Dudbridge argue that when many genome-wide variants are included the correlation among the genetic effects will tend to zero. We also assume no interactions in the genetic effects on the exposure, disease incidence, and progression - consistent with this we show in **Figure 10** that the genetic effects on BMI are similar in HF cases and controls.

The bias correction factor estimates for the CWBLS method using genome-wide independent SNPs at different minor allele frequency (MAF) inclusion thresholds are presented in **Figure 9**. Similar to the other instrument effect regression methods (Dudbridge 2019 & CWLS), the correction factor is influenced by the inclusion of unstable estimates from low-frequency variants but stabilizes when common variants (MAF >5%) are used. At a MAF inclusion threshold of >5%, the bias correction factor estimates for all-cause HF and the progression traits of all-cause mortality and the composite outcome were: 0.184 (95% CI: 0.068-0.30 and 0.492 (95% CI: 0.374-0.610), respectively.


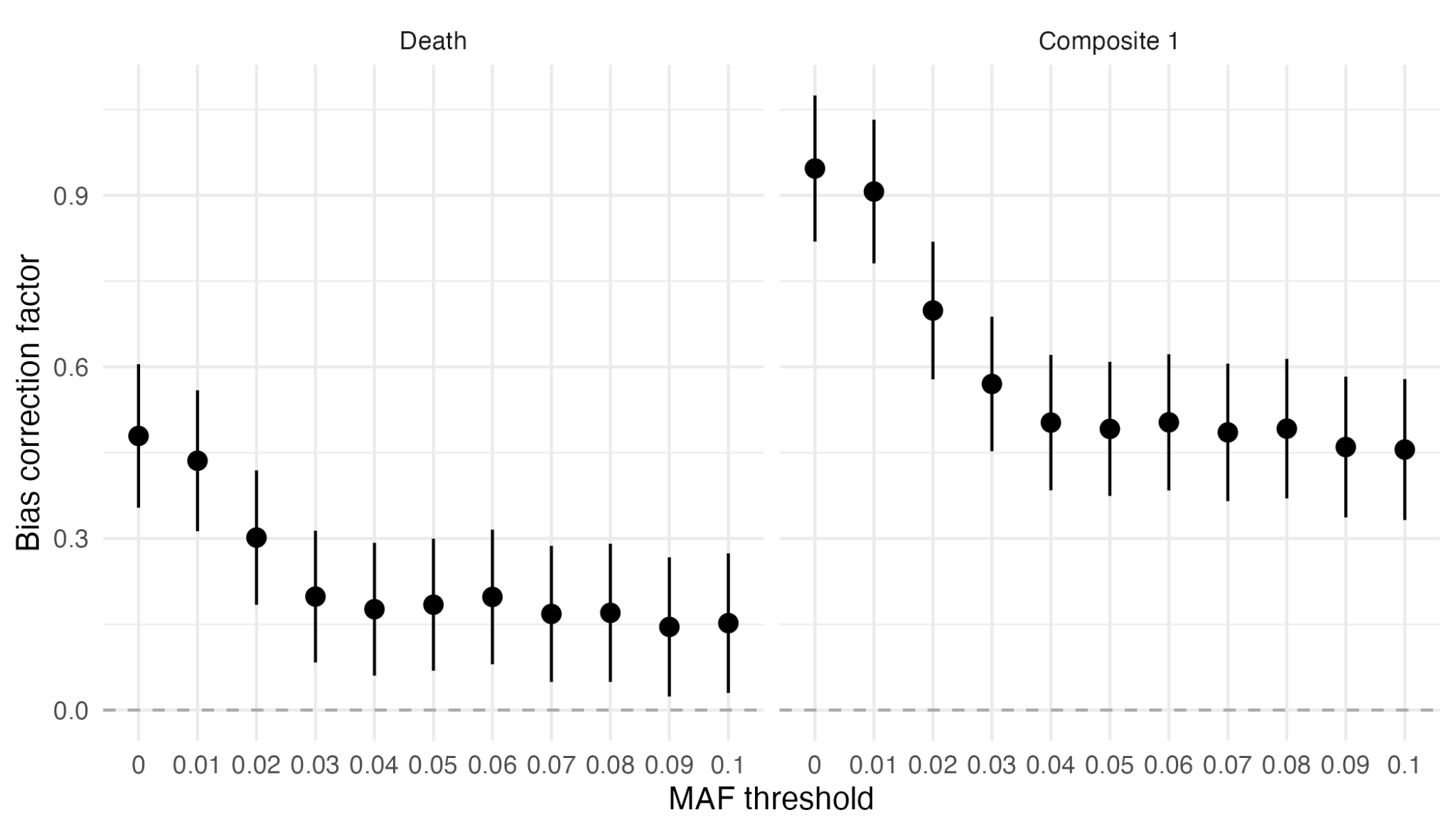


**Figure 9.** Bias correction factors for the all-cause mortality and composite endpoint progression traits in all-cause heart failure, obtained using the CWBLS Dudbridge method (R package *ColliderBias*), pruned genome-wide SNPs (*r2* = 0.1, *kb* = 250) with varying MAF inclusion threshold.

**Bias correction methods summary**

In summary, we applied the corrected weighted bivariate least squares (CWBLS) multivariable Mendelian randomization (MVMR) method as our primary approach to assess and adjust for index-event bias, given its recent development and ability to relax assumptions of causal independence between exposures (e.g., BMI), disease incidence, and progression while accounting for weak instruments. We present results from adjustments using the original Dudbridge (2019), corrected weighted least squares (CWLS), and standard two-sample Mendelian randomization (MR) methods as sensitivity analyses. For all instrument effect regression-based approaches (Dudbridge, CWLS, and CWBLS), we applied a minor allele frequency (MAF) threshold of >5% to ensure stability, as including low-frequency variants introduced noise and exaggerated regression dilution corrections. The Slope-Hunter method proved highly sensitive to minor variations in input parameters (e.g., P-value thresholds for incidence SNP selection) and was deemed unreliable in our low-heritability dataset, yielding unstable and contradictory correction factors.

**Figure 10 - BMI instrument variants in non-HF UKBB cohort vs. HF subset**

**
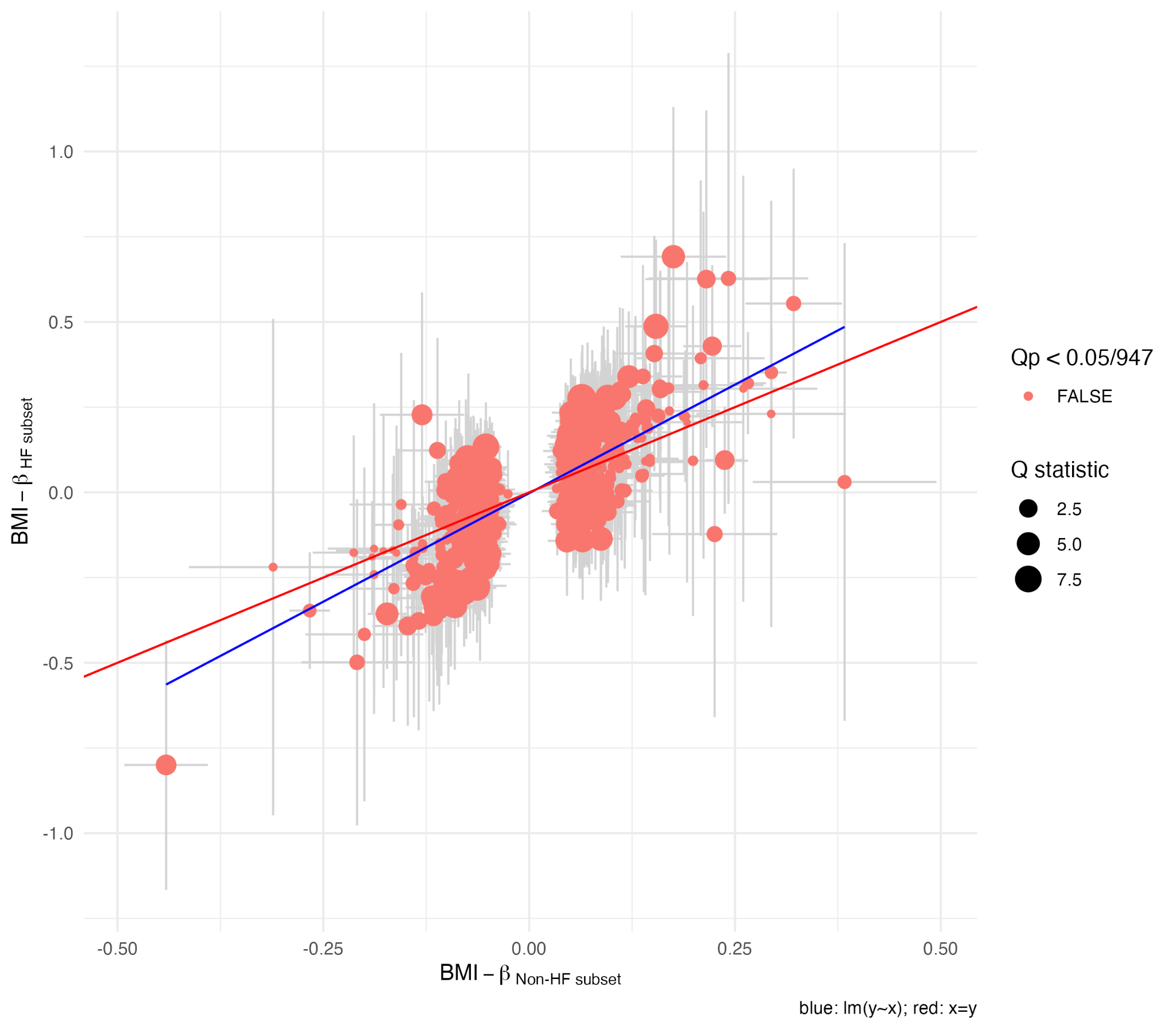
**

Genetic associations from GWAS of BMI in individuals in the UK-Biobank without heart failure (HF) (x-axis) versus individuals in the UK-Biobank with heart failure (y-axis). Data for the 947 variants used in the primary BMI genetic instrument are shown. No variants displayed significant evidence of effect heterogeneity between the non-HF and HF populations, confirming that the primary BMI instrument, derived from data from a general population, is valid for use in a HF only population. The red line represents the x=y line for perfect correlation, the blue line represents an unweighted linear regression slope.

**Figure 11 - Association Between Body Mass Index and Mortality: Observational and Mendelian Randomization Analyses**

**
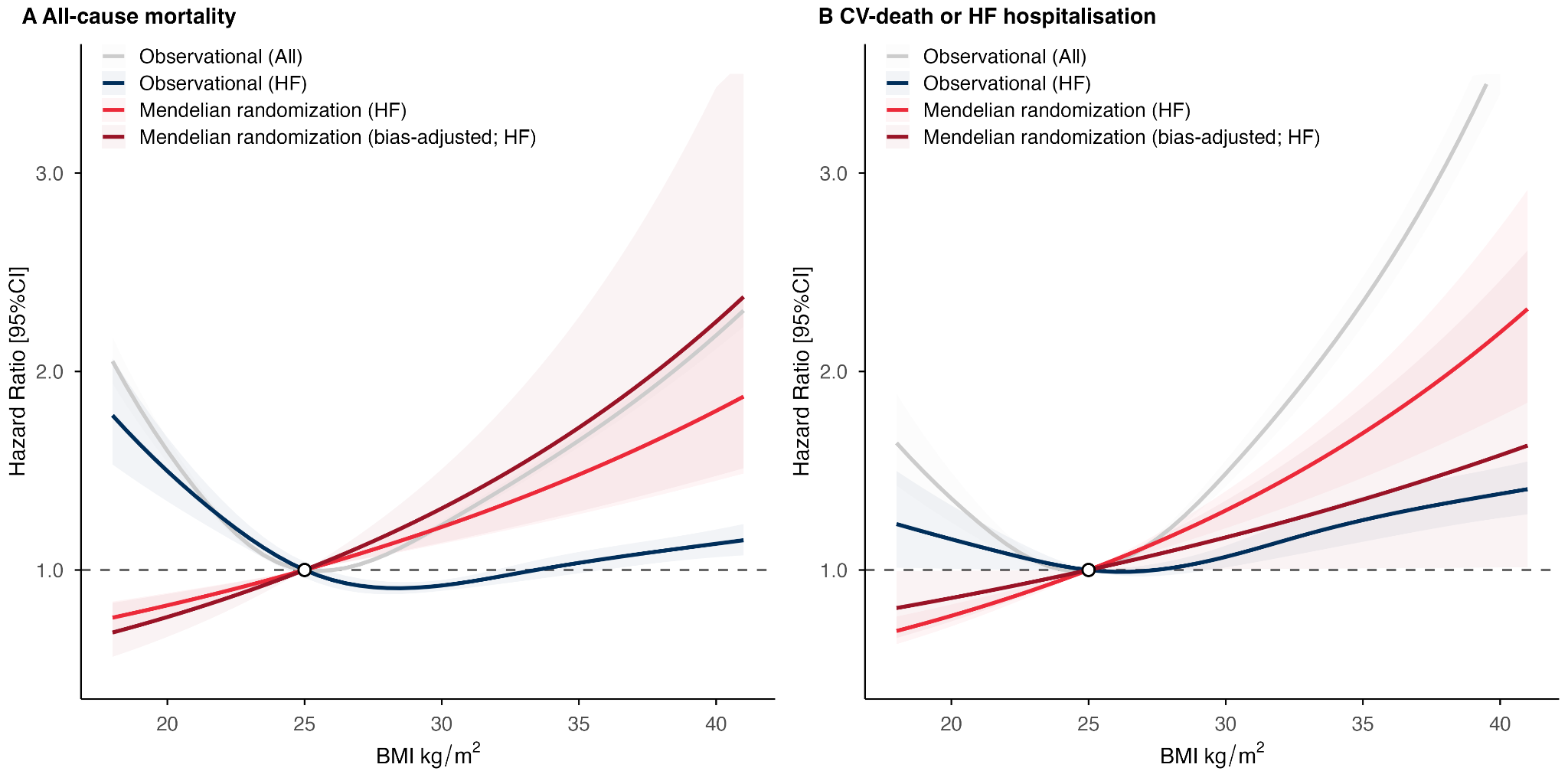
**

Association between body mass index (BMI) and clinical outcomes: (A) all-cause mortality and (B) cardiovascular death or heart failure (HF) hospitalization. Each panel presents data from two separate types of analysis: 1) observational BMI restricted cubic spline survival models using non-genetic data from either all individuals (grey curves), or individuals with HF (blue curves), within the UK Biobank; and 2) Mendelian randomization estimates of the association between BMI and HF outcomes, log-linearly extrapolated assuming a constant hazard ratio per standard deviation increase in genetically predicted BMI (red curves: unadjusted MR; dark red curves: bias-adjusted MR using the primary bias adjustment method, Bivariate MR CWBLS, from Cai and Dudbridge). Observational curves are derived from the models adjusted for age and sex, with estimates shown at the median age and for male sex, consistent with the covariate adjustment used in the GWAS of time-to-event outcomes. The observational analyses capture non-linear relationships between measured BMI and outcomes, while MR estimates assume a linear relationship on the log-hazard scale based on genetic instruments. All hazard ratios are referenced to BMI = 25 kg/m² (white circle). Shaded areas represent 95% confidence intervals.

**Figure 12 - Unadjusted MR estimates of BMI on HF and HF progression, multiple MR methods**


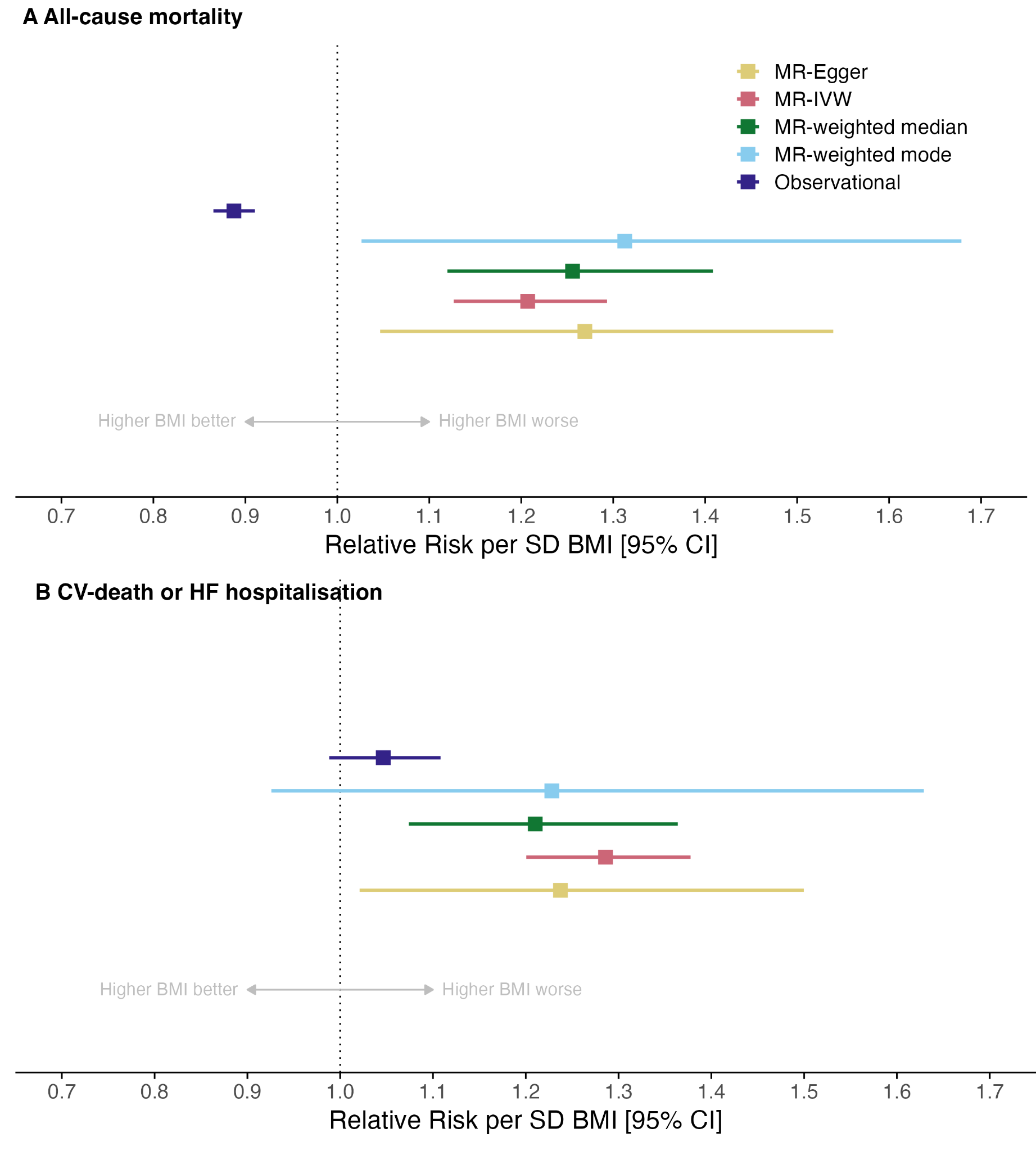


The plot presents the observational (dark blue) and unadjusted Mendelian randomisation estimates for the effect of a standard deviation increment in body mass index on heart failure clinical outcomes, estimated with multiple MR methods (colors). Error bars represent 95% CIs.

**Figure 13 - Unadjusted MR estimates of BMI on HF subtypes and HF progression, using multiple MR methods**


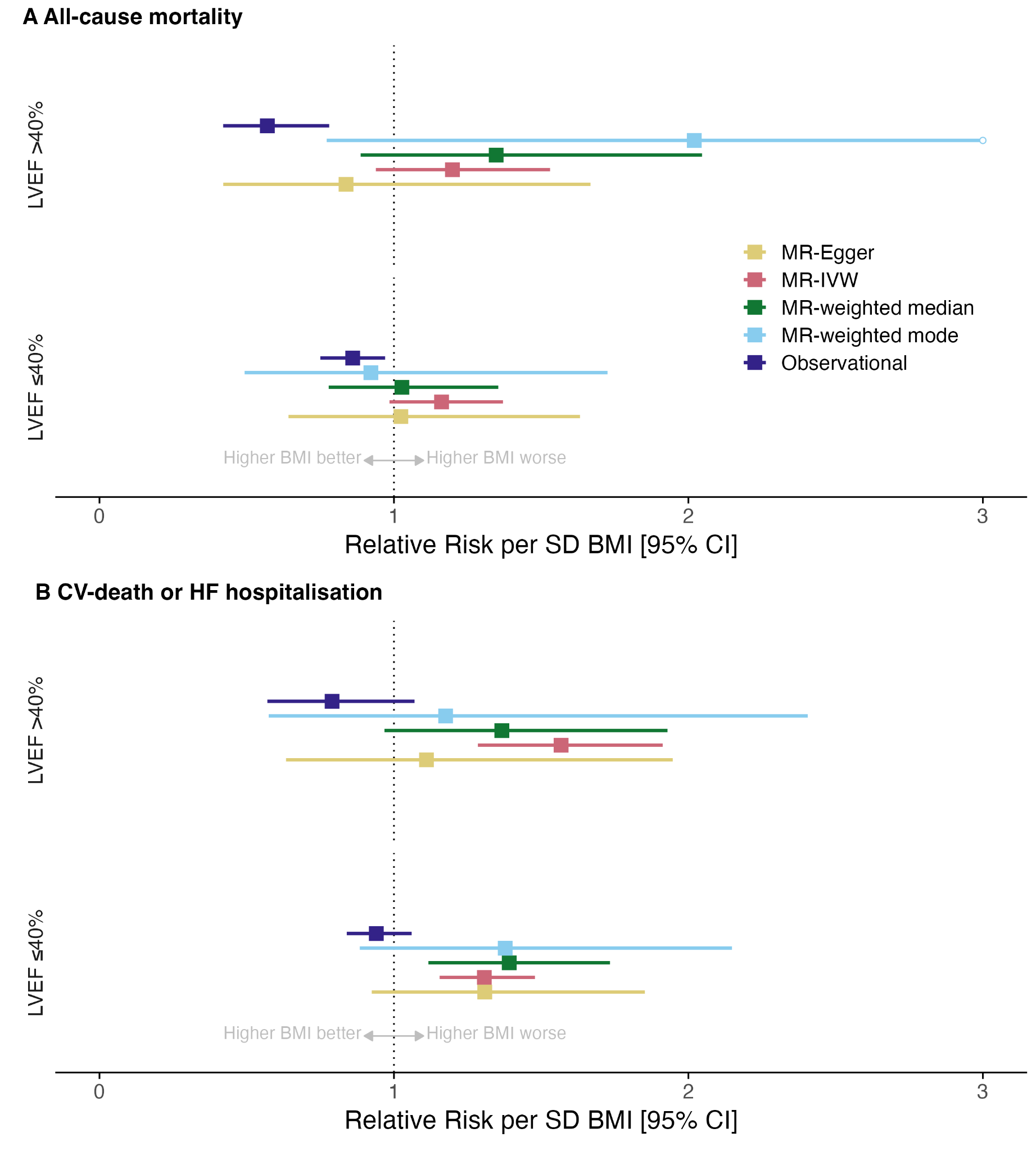


The plot presents the LVEF stratified observational (dark blue) and unadjusted Mendelian randomisation estimates for the effect of a standard deviation increment in body mass index on heart failure clinical outcomes, estimated with multiple MR methods (colours). Error bars represent 95% confidence intervals.

**Figure 14 - Bias-adjusted MR estimates of BMI on HF and HF progression, using multiple bias-adjustment methods**

**
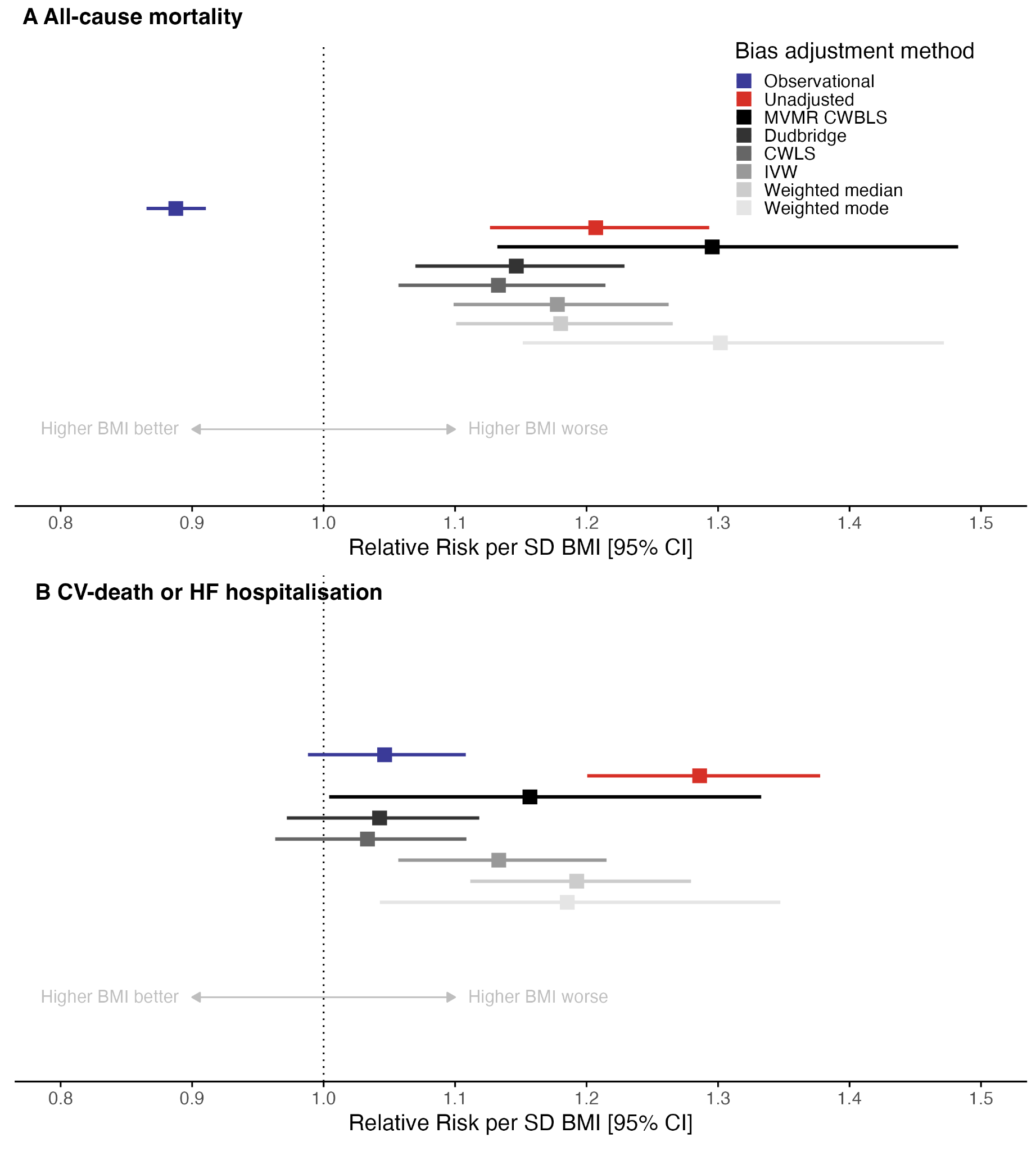
**

The plot presents the observational (dark blue), unadjusted (red), and bias-adjusted (shades of gray per bias-adjustment method) Mendelian randomization estimates for the effect of a standard deviation increment in body mass index on heart failure clinical outcomes. Error bars represent 95% CIs.

**Figure 15 - Bias-adjusted MR estimates of BMI on HF subtypes and HF progression, using multiple bias-adjustment methods**

**
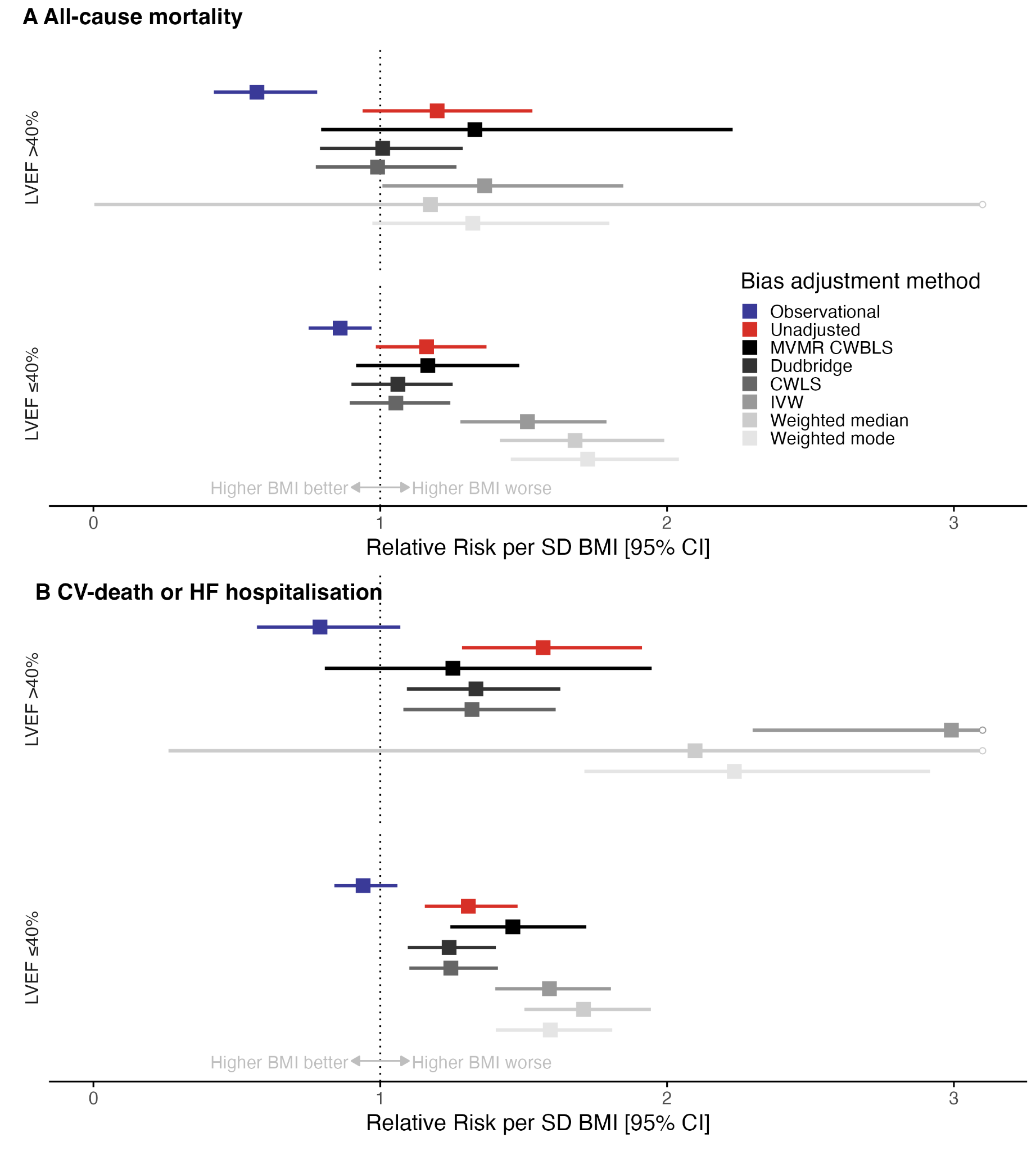
**The plot presents the LVEF stratified observational (dark blue), unadjusted (red), and bias-adjusted (shades of gray per bias-adjustment method) Mendelian randomization estimates for the effect of a SD increment in body mass index on heart failure clinical outcomes. Error bars represent 95% Cis.

**Figure 16 – Association of Genetic Scores with the Risk of All-cause Mortality in Individuals with Heart Failure (BMI instrument, excluding data from the UK Biobank)**


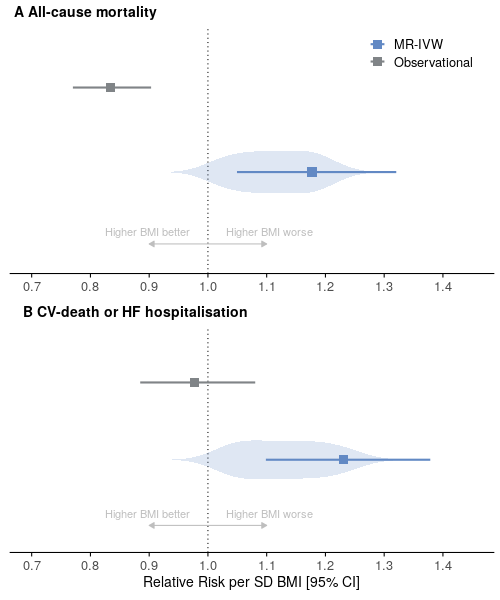


Panels A (all-cause mortality) and B (cardiovascular death or heart failure hospitalisation) show the change in hazard ratio per standard deviation increase in body mass index in individuals with diagnosed heart failure. Gray data points represent the association derived from observational data from the UK Biobank. Blue data points represent the association derived from genetic scores for body mass index. Bars indicate the 95% confidence interval. The shaded blue areas show the range of estimates resulting from the sensitivity analyses correcting for index event bias estimated with multiple methods and starting parameters. The BMI instrument was derived from a meta-analysis of the Genetic Investigation of Anthropometric Traits (GIANT, n = 235,069) consortium and Genetic Epidemiology Research in Adult Health and Aging cohort (GERA, n = 100,418) of predominantly European ancestry (183 variants), which does not contain individuals from the UK Biobank.[^9^](https://www.zotero.org/google-docs/?ApNbM3)

**References**

[1.](https://www.zotero.org/google-docs/?a9sQy7) [Dudbridge F, Allen RJ, Sheehan NA, et al. Adjustment for index event bias in genome-wide association studies of subsequent events. *Nat Commun*. 2019;10(1):1561. doi:10.1038/s41467-019-09381-w](https://www.zotero.org/google-docs/?a9sQy7)

[2.](https://www.zotero.org/google-docs/?a9sQy7) [Mitchell RE, Hartley AE, Walker VM, et al. Strategies to investigate and mitigate collider bias in genetic and Mendelian randomisation studies of disease progression. *PLOS Genet*. 2023;19(2):e1010596. doi:10.1371/journal.pgen.1010596](https://www.zotero.org/google-docs/?a9sQy7)

[3.](https://www.zotero.org/google-docs/?a9sQy7) [Cai S, Hartley A, Mahmoud O, Tilling K, Dudbridge F. Adjusting for collider bias in genetic association studies using instrumental variable methods. *Genet Epidemiol*. 2022;46(5-6):303-316. doi:10.1002/gepi.22455](https://www.zotero.org/google-docs/?a9sQy7)

[4.](https://www.zotero.org/google-docs/?a9sQy7) [Chang CC, Chow CC, Tellier LC, Vattikuti S, Purcell SM, Lee JJ. Second-generation PLINK: rising to the challenge of larger and richer datasets. *GigaScience*. 2015;4(1):s13742-015-0047-0048. doi:10.1186/s13742-015-0047-8](https://www.zotero.org/google-docs/?a9sQy7)

[5.](https://www.zotero.org/google-docs/?a9sQy7) [Mahmoud O, Dudbridge F, Davey Smith G, Munafo M, Tilling K. A robust method for collider bias correction in conditional genome-wide association studies. *Nat Commun*. 2022;13(1):619. doi:10.1038/s41467-022-28119-9](https://www.zotero.org/google-docs/?a9sQy7)

[6.](https://www.zotero.org/google-docs/?a9sQy7) [Bulik-Sullivan B, Finucane HK, Anttila V, et al. An atlas of genetic correlations across human diseases and traits. *Nat Genet*. 2015;47(11):1236-1241. doi:10.1038/ng.3406](https://www.zotero.org/google-docs/?a9sQy7)

[7.](https://www.zotero.org/google-docs/?a9sQy7) [Cai S, Allen RJ, Wain LV, Dudbridge F. Reassessing the association of MUC5B with survival in idiopathic pulmonary fibrosis. *Ann Hum Genet*. 2023;87(5):248-253. doi:10.1111/ahg.12522](https://www.zotero.org/google-docs/?a9sQy7)

[8.](https://www.zotero.org/google-docs/?a9sQy7) [Cai S, Dudbridge F. Estimating Causal Effects on a Disease Progression Trait Using Bivariate Mendelian Randomisation. *Genet Epidemiol*. 2025;49(1):e22600. doi:10.1002/gepi.22600](https://www.zotero.org/google-docs/?a9sQy7)

[9.](https://www.zotero.org/google-docs/?a9sQy7) [Hoffmann TJ, Choquet H, Yin J, et al. A Large Multiethnic Genome-Wide Association Study of Adult Body Mass Index Identifies Novel Loci. *Genetics*. 2018;210(2):499-515. doi:10.1534/genetics.118.301479](https://www.zotero.org/google-docs/?a9sQy7)
